# The Opa1-Dependent Mitochondrial Cristae Remodeling Pathway Controls Atrophic, Apoptotic, and Ischemic Tissue Damage

**DOI:** 10.1016/j.cmet.2015.05.007

**Published:** 2015-06-02

**Authors:** Tatiana Varanita, Maria Eugenia Soriano, Vanina Romanello, Tania Zaglia, Rubén Quintana-Cabrera, Martina Semenzato, Roberta Menabò, Veronica Costa, Gabriele Civiletto, Paola Pesce, Carlo Viscomi, Massimo Zeviani, Fabio Di Lisa, Marco Mongillo, Marco Sandri, Luca Scorrano

**Affiliations:** 1 Dulbecco Telethon Institute, Venetian Institute of Molecular Medicine, Via Orus 2, 35129 Padova, Italy; 2 Department of Biology, University of Padova, Via C. Colombo 3, 35121 Padova, Italy; 3 Department of Biomedical Sciences, University of Padova, Via C. Colombo 3, 35121 Padova, Italy; 4 Fondazione IRCCS Istituto Neurologico “C. Besta,” Via L. Temolo 4, 20126 Milan, Italy; 5 MRC Mitochondrial Biology Unit, MRC Building, Hills Road, Cambridge CB2 0XY, UK; 6 Institute of Neuroscience, National Research Council of Italy (CNR), Via C. Colombo 3, 35121 Padova, Italy; 7 Department of Cardiac, Thoracic and Vascular Sciences, University of Padova, Via Giustiniani, 2, 35128 Padova, Italy

## Abstract

Mitochondrial morphological and ultrastructural changes occur during apoptosis and autophagy, but whether they are relevant in vivo for tissue response to damage is unclear. Here we investigate the role of the optic atrophy 1 (OPA1)-dependent cristae remodeling pathway in vivo and provide evidence that it regulates the response of multiple tissues to apoptotic, necrotic, and atrophic stimuli. Genetic inhibition of the cristae remodeling pathway in vivo does not affect development, but protects mice from denervation-induced muscular atrophy, ischemic heart and brain damage, as well as hepatocellular apoptosis. Mechanistically, OPA1-dependent mitochondrial cristae stabilization increases mitochondrial respiratory efficiency and blunts mitochondrial dysfunction, cytochrome c release, and reactive oxygen species production. Our results indicate that the OPA1-dependent cristae remodeling pathway is a fundamental, targetable determinant of tissue damage in vivo.

## Highlights

• Mice with controlled Opa1 overexpression are viable and grow normally
• Opa1 protects from muscular atrophy, heart and brain ischemia, and liver apoptosis
• Opa1 reduces ROS production and cytochrome c release
• Opa1-controlled cristae remodeling is a targetable component of tissue damage

## Graphical Abstract

**Figure:**
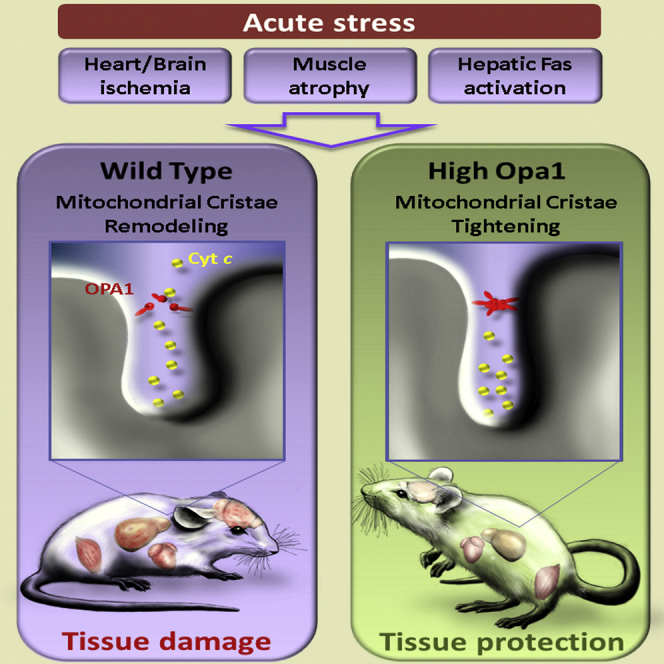

## Teaser

Varanita et al. show that a mouse model of controlled overexpression of the master mitochondrial cristae biogenetic factor Opa1 resists skeletal muscle atrophy, heart and brain ischemic damage, and massive liver apoptosis by blunting mitochondrial dysfunction and cytochrome c release. Together with the accompanying paper by Civiletto et al., the authors offer a proof of principle of the potential for therapies targeting cristae remodeling in sporadic and genetic diseases.

## Introduction

Mitochondria are crucial organelles in energy conversion (Danial et al., 2003; Rizzuto et al., 2000), and, not surprisingly, impaired mitochondrial function affects organs where energy demand is high, like heart, skeletal muscle, and brain (DiMauro and Schon, 2003). Cellular damage is not only the consequence of mitochondrial bioenergetic failure, given that these organelles also play a crucial role in apoptosis, when in response to several stimuli they release cytochrome c and other pro-apoptotic proteins that execute cell demise (Wang, 2001). Mitochondrial permeabilization during apoptosis is controlled by members of the Bcl-2 family and is accompanied by both morphological and ultrastructural changes of the organelle (Danial and Korsmeyer, 2004; Wasilewski and Scorrano, 2009). Mitochondrial network fragmentation and cristae remodeling with widening of cristae junctions are both required for the complete release of cytochrome c (Scorrano et al., 2002; Yamaguchi et al., 2008).

A family of dynamin-related large GTPases controls mitochondrial fusion and fission (Griparic and van der Bliek, 2001). Fission occurs upon the recruitment of dynamin-related protein 1 (DRP1) on the outer mitochondrial membrane (OMM) (Cereghetti et al., 2008), where it binds to its adaptors, including fission 1 (Fis1), mitochondrial fission factor (MFF), and mitochondrial division (Mid) 49 and 51 (Palmer et al., 2011). Mitochondrial fusion is controlled by mitofusins (MFN) 1 and 2 in the OMM and by OPA1 in the inner mitochondrial membrane (IMM) (Chen et al., 2003; Santel et al., 2003; Cipolat et al., 2004). The IMM can be divided in two subcompartments: the so-called “boundary membrane” and the cristae, separated from the former by narrow tubular junctions (Frey and Mannella, 2000). Upon activation of mitochondrial respiration, cristae transition from the orthodox to the condensed morphology (Hackenbrock, 1966), and during apoptosis they remodel in response to pro-apoptotic BH3-only BCL-2 family members such as BID, BIM-S, and BNIP3, independently from outer membrane permeabilization (Scorrano et al., 2002; Yamaguchi et al., 2008; Landes et al., 2010). In addition to, and independently from, its role in mitochondrial fusion, OPA1 regulates apoptotic cristae remodeling by forming oligomers that participate in cristae junction formation and maintenance (Frezza et al., 2006; Cipolat et al., 2006). Moreover, OPA1, by maintaining mitochondrial cristae morphology, has a direct metabolic effect, stabilizing respiratory chain supercomplexes (RCSs) (Cogliati et al., 2013).

The identification of the key role of OPA1 in cristae remodeling suggests a potential approach to manipulate this process in vivo; however, constitutive as well as conditional tissue-specific *Opa1* ablation in the mouse is lethal (Zhang et al., 2011; Davies et al., 2007), and uncontrolled OPA1 overexpression is toxic (Cipolat et al., 2004), complicating the generation of suitable in vivo models. To circumvent these difficulties, we recently generated a mouse model where a transgene carrying *Opa1* isoform 1 under the control of the ubiquitous human β-actin promoter was purposely targeted to a permissive X chromosome region, without altering endogenous gene expression (Cogliati et al., 2013). We capitalized on this mouse model to investigate the role of the OPA1-dependent cristae remodeling pathway in vivo. Our data indicate that mild OPA1 overexpression is compatible with life and blunts damage of highly metabolically active organs in response to apoptotic, necrotic, and atrophic stimuli by reducing cytochrome c release and mitochondrial dysfunction, thereby highlighting the importance of cristae shape in tissue homeostasis and non-developmental cell death.

## Results

### Controlled OPA1 Overexpression Is Compatible with Embryonic Development and Does Not Affect Lifespan

**Figure 1:**
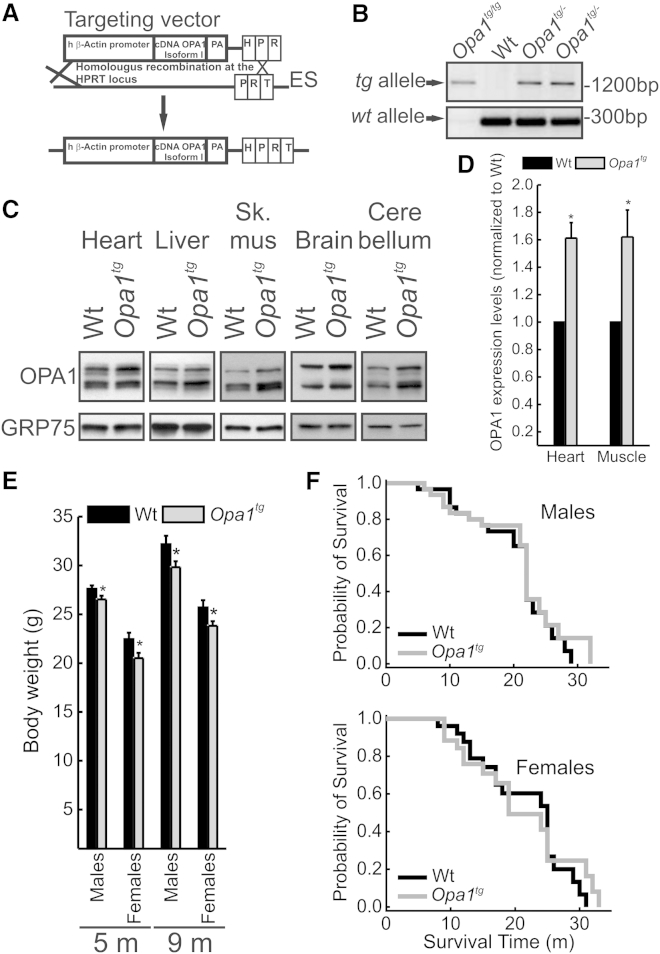
*Opa1*^*tg*^ Mice Are Viable, Fertile, and Grow Normally (A) Schematic representation of targeted transgenesis strategy into the permissive hypoxanthine phosphoribosyltransferase (HPRT) locus. The targeted vector includes a 5′ HPRT homology arm, β-actin promoter, the transgene cDNA OPA1 isoform 1, and 3′ HPRT homology arm including a part of HPRT promoter and a homologous region to the mouse HPRT locus. The targeting construct is linearized before electroporation into BPES cells. Homologous recombination of the 3′ arm reconstitutes a functional HPRT gene, allowing the selection of targeted BPES cells on stringent hypoxanthine-aminopterin-thymidine (HAT) conditions. (B) PCR analysis of genomic DNA from wild-type (WT), heterozygous (*Opa1*^+/−^), and homozygous (*Opa1*^+/+^) *Opa1*^*tg*^ mouse tail. (C) Equal amounts of protein from tissues of the indicated genotypes were separated by SDS-PAGE and immunoblotted with the indicated antibodies. (D) Densitometric analysis of OPA1 protein level in tissue from heart and muscle. Data represent average ± SEM (n = 7 for each group). ^∗^p < 0.05 in an unpaired two-sample Student’s t test. (E) Average ± SEM body weight of 5-month-old male (n = 34 for each group) and female (n = 13 for each group) and of 9-month-old male (n = 18 for each group) and female (n = 16 for each group) C57/Bl6 littermates of the indicated genotype. ^∗^p < 0.05 in a two-tailed ANOVA test. (F) Kaplan-Meier censorial analysis of C57/Bl6 mice of the indicated genotype (n = 30 male and n = 26 female littermates). See also Figure S1.

*Opa1*-overexpressing mice (*Opa1*^*tg*^) generated by targeting a single copy of murine *Opa1* isoform 1 driven by human β actin promoter immediately upstream of the mouse X chromosome *Hprt* locus (Cogliati et al., 2013) (Figures 1A and 1B) were backcrossed for more than 10 generations in C57/Bl6J and SV129 genetic backgrounds. *Opa1*^*tg*^ mice were born at the expected Mendelian frequency, developed and grew normally, and were apparently indistinguishable from sex- and age-matched wild-type (WT) littermates. The expected mild (∼1.5-fold), but statistically significant, OPA1 overexpression was detected by western blot analysis in all the tissue tested (Figures 1C and 1D). Conversely, expression levels of the key mitochondrial fusion-fission proteins as well as of the pro- and anti-apoptotic Bcl-2 family proteins were unaltered (Figures S1A and S1B). While *Opa1*^*tg*^ and WT littermates’ body weight was comparable during the first 60 days after birth (Figure S1C), in the obesity-prone C57/Bl6J strain (Almind and Kahn, 2004) the body weight of 5- to 9-month-old *Opa1*^*tg*^ individuals was slightly, but significantly, reduced in respect to their WT littermates (Figure 1E). This effect of OPA overexpression was not recorded in the Sv129 background (Figure S1D) and had no effect (beneficial or detrimental) on the overall survival as shown by a Kaplan-Meyer censorial analysis (Figures 1F and 1G). In conclusion, *Opa1*^*tg*^ mice are viable and display no apparent major phenotype, indicating that a mild OPA1 overexpression is compatible with life and fertility.

### Physiological Heart Hypertrophy in 9-Month-Old *Opa1*^*tg*^ Mice

**Figure 2:**
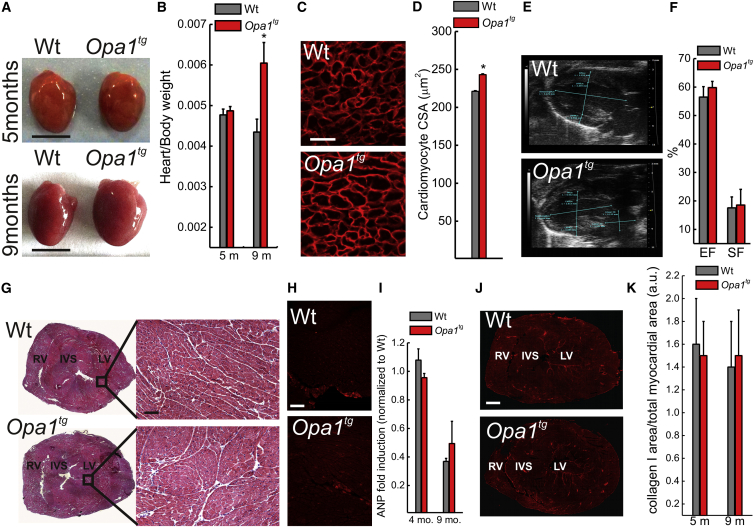
*Opa1*^*tg*^ Mice Develop a Non-pathological Cardiac Hypertrophy (A) Representative photographs of hearts dissected from littermates of the indicated genotype and age. Scale bars, 0.5 cm. (B) Average ± SEM heart/body weight ratio in littermates of the indicated genotype and age (n = 8 in each group). ^∗^p < 0.05 in a two-tailed ANOVA test versus 5 months *Opa1*^*tg*^ dataset. (C) Representative detail confocal images of left ventricular cryosections stained for dystrophin from 9-month-old mice of the indicated genotype. Scale bar, 25 μm. (D) Experiments were as in (C). Data represent mean ± SEM of four independent experiments. ^∗^p < 0.05 in an unpaired two-sample Student’s t test. (E) Echocardiographic long axis view of hearts from 9-month-old littermates of the indicated genotype. LV, left ventricle; A, aorta. (F) Experiments were as in (E). Data represent mean ± SEM percentage of cardiac ejection fraction (EF) and shortening (SF) of four independent experiments. (G) H&E staining of ventricular cryosections from 9-month-old littermates of the indicated genotype. Scale bars, 100 μm. (H) Representative immunofluorescence images of ventricular cryosections stained for atrial natriuretic peptide (ANP) from 9-month-old littermates of the indicated genotype. Scale bar, 100 μm. (I) Data represent average ± SEM of ANP mRNA levels determined by RT-PCR in 4- and 9-month-old littermates of the indicated genotype (n = 4 for each group). (J) Representative immunofluorescence images of ventricular cryosections stained for collagen I from 9-month-old littermates of the indicated genotype. Scale bar, 100 μm. (K) Experiments were as in (J). Data represent mean ± SEM of four independent experiments. See also Figure S2.

In order to address the long-term effects of OPA1 overexpression, we aged the *Opa1*^*tg*^ animals and analyzed morphologically and histologically several organs at different time points. Upon gross inspection, hearts of 9-month-old *Opa1*^*tg*^ mice appeared bigger than their WT littermates (Figure 2A). Accordingly, the heart/body weight ratio (Figure 2B) and the cardiomyocyte cross-sectional area (Figures 2C and 2D) were increased in 9-month-old *Opa1*^*tg*^ mice, but not in younger animals (Figures S2A and S2B). Since other parenchymal organs, such as liver and kidney, were not enlarged, as revealed by post-mortem as well as in vivo echography inspection (Figures S2D–S2F), we concluded that the hypertrophy was specific to the heart. The observed hypertrophy was not accompanied by echocardiographic signs of cardiac dysfunction at 9 (Figures 2E and 2F) as well as at 5 (Figure S2G) months of age. Echocardiographic speckle tracking analysis to assess myocardial performance/load relationship excluded that *Opa1*^*tg*^ hearts contract against an increased afterload, indicative of increased blood pressure, a major cause of heart hypertrophy: the time to peak (TtPk) of myocardial shortening of longitudinal and radial strains were comparable in *Opa1*^*tg*^ and WT hearts (longitudinal strain TtPk, 81.6 ± 6.15 ms in *Opa1*^*tg*^ versus 63.0 ± 4.1 ms in WT; radial strain TtPk, 50.0 ± 33.4 ms in *Opa1*^*tg*^ versus 49.3 ± 20.1 ms in WT). All the tests for pathological hypertrophy were negative: histology of 9-month-old WT and *Opa1*^*tg*^ hearts was superimposable (Figure 2G), the fetal hypertrophy gene atrial natriuretic peptide (ANP) (Du, 2007) was not induced, as judged by immunofluorescence and RT-PCR analysis (Figures 2H and 2I), and hearts were also negative for the other hypertrophy gene β-myosin heavy chain (β-MHC) (Figure S2H). Furthermore, the lack of collagen-I immunostaining excluded interstitial fibrosis (Figures 2J and 2K) in 9-month-old *Opa1*^*tg*^ hearts. Finally, since mitochondrial dysmorphology and dysfunction characterize pathological cardiac hypertrophy (Abel and Doenst, 2011), we set out to investigate mitochondrial shape and activity in 9-month-old *Opa1*^*tg*^ hearts. Mitochondrial ultrastructure, evaluated by electron microscopy (EM; Figure S2I), biogenesis, measured by real-time PCR analysis of mRNA levels of adenine translocase 1 (ANT1), controlled by the master biogenetic factor PGC1α (Figure S2J), as well as bioenergetics, indicated by biochemical and histological respiratory chain complexes (RCCs) activity measurements (Figures S2K and S2L), were not impaired. Complex III and IV activities were in fact increased, probably as a consequence of RCS stabilization by OPA1 (Cogliati et al., 2013). Thus, 9-month-old *Opa1*^*tg*^ animals display physiological heart hypertrophy, and indeed they perform slightly, but significantly, better than their WT littermates on a treadmill exercise test (Civiletto et al., 2015 [this issue of *Cell Metabolism*]).

### *Opa1*^*tg*^ Mice Are Protected from Denervation-Induced Muscular Atrophy

**Figure 3:**
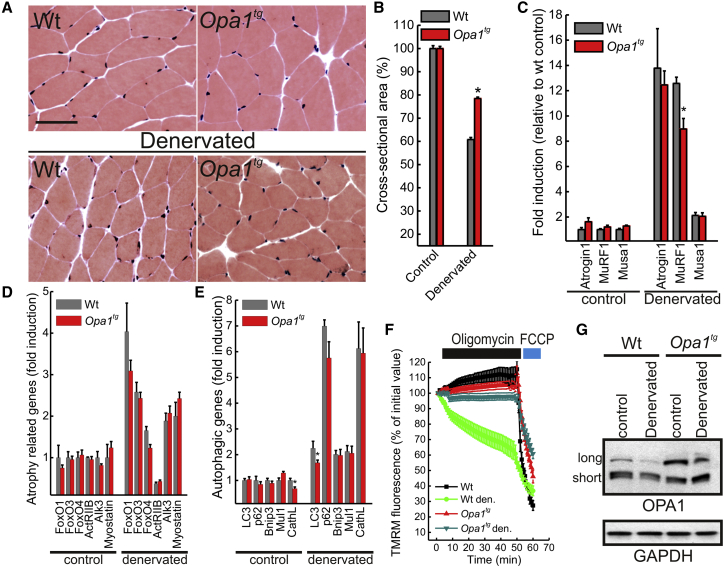
*Opa1*^*tg*^ Mice Are Protected from Denervation-Induced Muscle Atrophy (A) H&E staining of control and 10-days-denervated gastrocnemial cryosections from 9-month-old littermates of the indicated genotype. Scale bars, 50 μm. (B) Experiments were as in (A). Muscle loss 10 days after denervation was quantified by cross-sectional area (CSA) measurement of innervated and denervated fibers. Data represent mean ± SEM of five independent experiments. ^∗^p < 0.05 in an unpaired two-sample Student’s t test. (C–E) Data represent average ± SEM of the indicated mRNA levels determined by RT-PCR in littermates of the indicated genotype treated as indicated (n = 5 for each group). ^∗^p < 0.05 in an unpaired two-sample Student’s t test. (F) Data are average ± SEM of five independent experiments of TMRM fluorescence analysis over mitochondrial regions in fibers isolated from FDB of the indicated genotype treated as indicated. (G) Equal amounts (30 μg) of protein from gastrocnemius isolated from littermates of the indicated genotype treated as indicated were separated by SDS-PAGE and immunoblotted using the indicated antibodies. Den., denervated. See also Figure S3.

Since cardiomyocyte size results from the balance between trophic and atrophic processes, we wished to investigate if OPA1 overexpression counteracted the atrophic program that in muscle activates autophagy and requires mitochondrial fragmentation (Romanello et al., 2010) and in heart is exacerbated during aging when it is accompanied by lipofuscinosis (De Meyer et al., 2010). We therefore turned to ischiectomy-induced muscle loss, an established model of muscle atrophy where mitochondria are targets of degradation and active participants in myonuclear apoptosis (Siu and Alway, 2005; Sandri et al., 2006; Adhihetty et al., 2007). Histological analysis did not reveal signs of inflammation, degeneration, or regeneration in gastrocnemius sections from control or 10-day-denervated 5-month-old WT and *Opa1*^*tg*^ mice (Figure 3A), but *Opa1*^*tg*^ mice were significantly protected from denervation-induced muscle mass loss (Figure 3B) in both oxidative or glycolytic fibers (Figure S3A). Accordingly, induction of Muscle Ring-Finger protein 1 (Murf1), the muscle-specific atrophy-related ubiquitin ligase that controls degradation of sarcomeric proteins (Sandri, 2013), was reduced in denervated *Opa1*^*tg*^ muscles (Figure 3C), suggesting that OPA1 overexpression inhibits the atrophic program. Mechanistically, however, expression levels of atrophy-related genes of the ubiquitin-proteasome and autophagy-lysosome families that control myofiber size during disuse muscle atrophy (Powers et al., 2007; Sandri, 2008) were comparable in control and denervated WT and *Opa1*^*tg*^ muscles (Figures 3D and 3E), and autophagic flux was not altered in mouse adult fibroblasts (MAFs) derived from *Opa1*^*tg*^ mice (Figures S3B and S3C). We therefore investigated mitochondrial biogenesis and function in control and denervated muscles. Levels of the mitochondrial dynamics genes (except, of course, Opa1) and of the mitochondriobiogenetic factor Pgc1α were not changed (Figure S3D), suggesting that OPA1 overexpression does not induce biogenesis. Conversely, an established assay of mitochondrial dysfunction (Irwin et al., 2003) that characterizes muscle atrophy (Seo et al., 2010) revealed that *Opa1*^*tg*^ fibers were protected from denervation-induced mitochondrial dysfunction (Figure 3F). This assay reveals latent mitochondrial dysfunction and was more sensitive than histological and biochemical cytochrome c oxidase (COX) activity measurements, which resulted normal in gastrocnemius (Figures S3E and S3F) and in soleus (Figure S3G) in both genotypes, also after denervation. We could correlate the observed protection to the levels of the long OPA1 form that supports most of OPA1 functions (Anand et al., 2014) and that was almost completely degraded in denervated WT, but not *Opa1*^*tg*^, muscles (Figure 3G; long OPA1 represented only ∼4% of total OPA1 in WT, but ∼17% in *Opa1*^*tg*^-denervated muscles). Notably, the protection from muscular atrophy was long term, as shown by the preservation of muscular function and mass by OPA1 overexpression in a severe model of myopathy caused by skeletal muscle COX 15 (*Cox15*^*sm*/sm^) ablation (see the accompanying manuscript by Civiletto et al. [2015] [this issue of *Cell Metabolism*]). Thus, OPA1 overexpression protects from acute and chronic muscle atrophy by blunting mitochondrial dysfunction and expression of the critical ubiquitin ligase MuRF1, without altering the autophagic program or inducing mitochondrial biogenesis.

### *Opa1*^*tg*^ Mice Are Protected from Ischemic Damage

**Figure 4:**
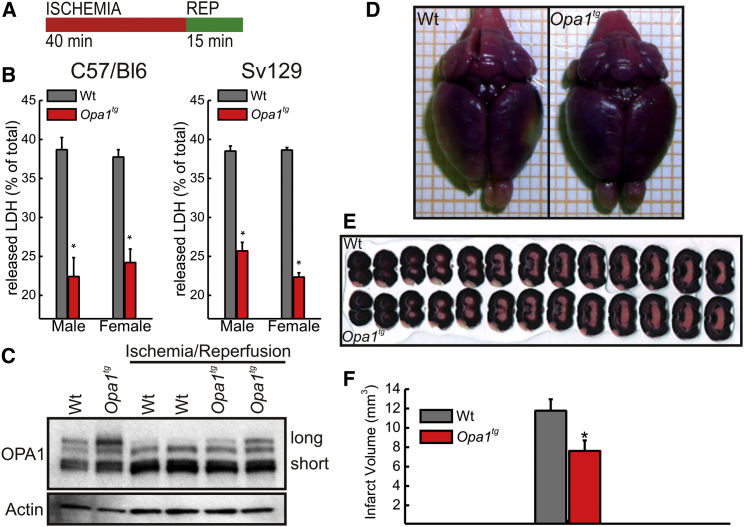
*Opa1*^*tg*^ Mice Are Protected from Ischemic Damage (A) Schematic representation of the experimental ischemia-reperfusion protocol used. (B) Data represent average ± SEM of eight independent experiments of lactate dehydrogenase (LDH) release during reperfusion in 5-month-old littermates of the indicated genotype, sex, and background (n = 8 for each group). ^∗^p < 0.05 in a two-tailed ANOVA test. (C) Equal amounts (30 μg) of heart protein from littermates of the indicated genotype treated as indicated were separated by SDS-PAGE and immunoblotted using the indicated antibodies. (D) Representative images of TTC-stained whole brains from 3-month-old Sv129 female littermates of the indicated genotype mice 72 hr after middle cerebral artery occlusion (MCAo). (E) Representative TTC-stained 250-μm brain sections from 3-month-old Sv129 female littermates of the indicated genotype 72 hr after MCAo. Slices are aligned from anterior to posterior. (F) Data represent average ± SEM of four independent experiments carried out as in (E). ^∗^p < 0.05 in an unpaired two-sample Student’s t test.

The protection of *Opa1*^*tg*^ mice from muscular atrophy prompted us to investigate other models of tissue damage where mitochondria play a central role. Myocardial infarction, besides being the leading cause of death in the Western world, is characterized by central necrosis and peripheral apoptosis, two processes in which mitochondrial dysfunction participates during both the ischemic and the reperfusion phases (Murphy and Steenbergen, 2008). By using a classical Langendorff-perfused heart preparation, we subjected hearts of 5-month-old mice to 40 min of ischemia followed by 15 min of reperfusion (I/R; Figure 4A) (Barbato et al., 1996) and assessed cardiac injury by measuring lactate dehydrogenase (LDH) release during the reperfusion period. OPA1 overexpression reduced the amount of released LDH in male and female C57/Bl6 and SV129 mice (Figure 4B), thereby excluding potentially confounding effects of genetic background and gender that influence the response to heart ischemia (Du, 2004). Similar to what was observed during skeletal muscle denervation, I/R was accompanied by long OPA1 degradation in WT, but not *Opa1*^*tg*^, hearts (Figure 4C; long OPA1 represents only ∼0.6% of total OPA1 in WT and ∼10% in *Opa1*^*tg*^ hearts). The protection from ischemia was not confined to heart, since infarct volume, assessed in triphenyl tetrazolium chloride (TTC)-stained serial sections from brains 3 days after middle cerebral artery occlusion (MCAo) (Colak et al., 2011), was significantly reduced in *Opa1*^*tg*^ mice (Figures 4D–4F). These results indicate that mild OPA1 overexpression protects heart and brain from ischemic damage.

### *Opa1*^*tg*^ Are Less Susceptible to Fas-Induced Liver Damage

**Figure 5:**
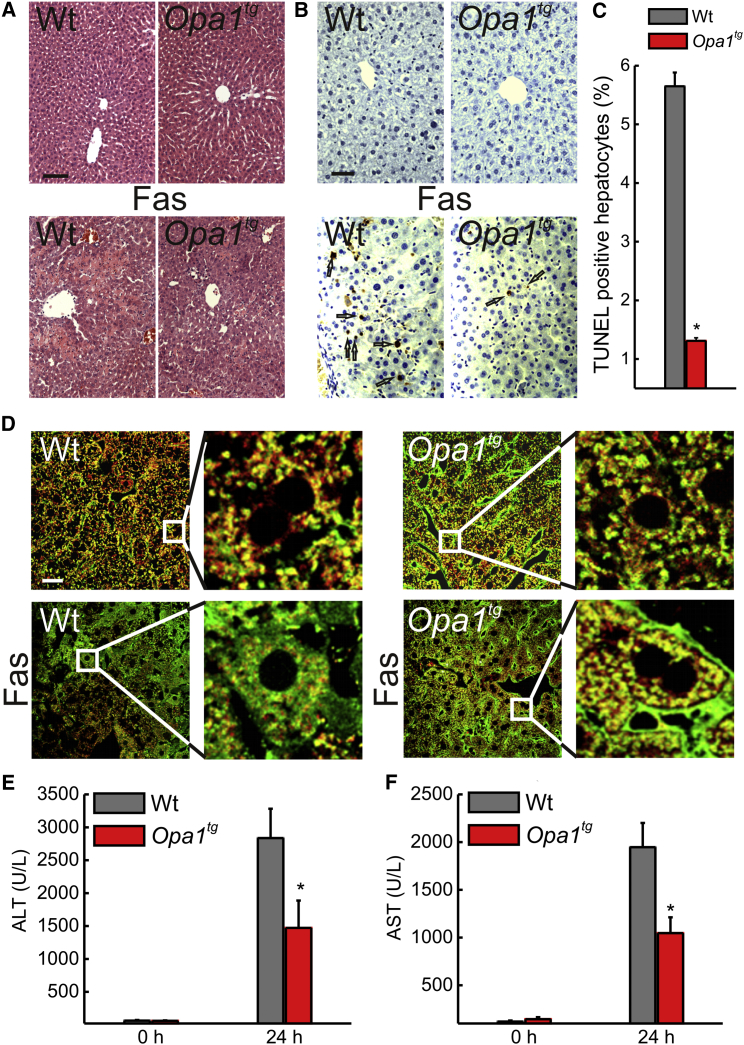
*Opa1*^*tg*^ Mice Are Protected from Fas-Induced Apoptotic Liver Damage (A) Representative images of H&E-stained paraffin-embedded liver sections from littermates of the indicated genotype. Where indicated mice were tail vein injected with 0.25 μg/g anti-Fas antibody (Fas), sacrificed after 24 hr, and livers were explanted for histology. Scale bar, 50 μm. (B) Experiments were as in (A), except that paraffin-embedded liver sections were TUNEL stained. Arrows indicate TUNEL-positive hepatocytes. Scale bar, 25 μm. (C) Data represent average ± SEM of four independent experiments carried out as in (B). At least 30 images per group per experiment were analyzed. ^∗^p < 0.05 in an unpaired two-sample Student’s t test. (D) Representative confocal images of liver cryosections from littermates of the indicated genotypes treated as indicated and immunostained for cytochrome c (green) and TOM20 (red). The boxed areas are magnified 5×. Scale bar, 20 μm. (E and F) Plasma ALT (E) or AST (F) levels in mice of the indicated genotypes at the indicated time points after tail vein injection of 0.25 μg/g anti-Fas antibody. Data represent average ± SEM (n = 7 in E, and n = 8 in F, for each group). ^∗^p < 0.05 in an unpaired ANOVA test. See also Figure S4.

The protection afforded by OPA1 from heart and brain ischemia corroborates early findings that implicated OPA1 and OPA1-dependent cristae remodeling in cell death. However, we wished to further challenge this hypothesis by turning to a model of in vivo apoptosis. Fas receptor activation in the liver by means of tail vein injection of anti-Fas-activating antibodies is a well-characterized model of severe apoptotic liver damage (Ogasawara et al., 1993) where Fas-induced death is dependent on mitochondria (Yin et al., 1999; Wei et al., 2000). While liver histology was not different in 3-month-old BL6 WT and *Opa1*^*tg*^ mice, 24 hr after tail vein injection of an activating anti-Fas antibody, damage was greatly decreased in *Opa1*^*tg*^ livers, where the lobular architecture was preserved and the hemorrhagic infiltrates reduced (Figure 5A). Accordingly, TUNEL staining revealed a significant reduction in apoptotic cell death in *Opa1*^*tg*^ livers (Figures 5B and 5C). Also, Sv129 *Opa1*^*tg*^ mice were less susceptible than their WT littermates to the same anti-Fas antibody-induced liver damage, as judged by survival and liver histology (Figure S4). Mechanistically, the protection afforded by OPA1 correlated with the inhibition of cytochrome c release from mitochondria, as revealed by the analysis of subcellular cytochrome c distribution in Fas-treated WT and *Opa1*^*tg*^ livers (Figure 5D). Consistently, the increase in plasma alanine and aspartate transaminases (ALT and AST) levels, a liver damage indicator, was significantly lower in Fas-treated *Opa1*^*tg*^ mice versus their WT-treated littermates (Figures 5E and 5F). Thus, OPA1 overexpression inhibits mitochondrial-dependent hepatocyte apoptosis in vivo.

### *Opa1*^*tg*^ Mitochondria Are Resistant to Cristae Remodeling and Cytochrome C Release

**Figure 6:**
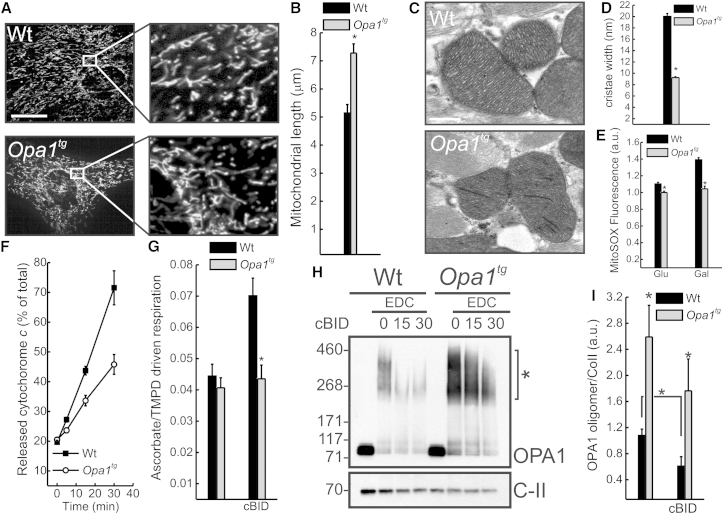
*Opa1*^*tg*^ Mitochondria Are Resistant to Apoptotic Cristae Remodeling and Cytochrome C Release (A) Representative confocal images of mitochondrially targeted YFP transfected in primary myoblasts of the indicated genotype. Scale bar, 20 μm. (B) Data represent average ± SEM of four independent experiments (30 cells/experiment) of mitochondrial major axis length calculation in experiments as in (A). ^∗^p < 0.05 in an unpaired two-sample Student’s t test. (C) Representative electron micrographs of hearts of the indicated genotype. Scale bar, 500 nm. (D) Morphometric analysis of cristae width in 80 randomly selected mitochondria of 5-month-old hearts from WT and *Opa1*^*tg*^ mice. ^∗^p < 0.05 in an unpaired two-sample Student’s t test. (E) Fold increase in MitoSOX fluorescence after treatment with antimycin A (10 μM, 20 min) in cells incubated for 24 hr in DMEM supplemented with the indicated monosaccharides. Data represent average ± SEM of four independent experiments. ^∗^p < 0.05 in an unpaired two-sample Student’s t test. (F) Mitochondria isolated from livers of the indicated genotypes were treated for the indicated times with 40 pmol/mg cBID, and cytochrome c release was measured by ELISA. Data represent average ± SEM of five independent experiments. (G) Ascorbate/TMPD-driven respiration of purified liver mitochondria of the indicated genotypes treated where indicated for 15 min with cBID. Data represent average ± SEM of four independent experiments. ^∗^p < 0.05 in an unpaired two-sample Student’s t test. (H) Purified muscle mitochondria of the indicated genotype were treated for the indicated times with cBID, and, where indicated, 10 mM EDC was added. Equal amounts (30 μg) of proteins were separated by SDS-PAGE and immunoblotted using the indicated antibodies. (I) Densitometric analysis of OPA1 oligomers. Experiments were as in (H). Data represent average ± SEM of five independent experiments. ^∗^p < 0.05 in a two-way ANOVA with the corresponding time point WT or between the indicated samples. See also Figure S5.

We next wished to address mechanistically how OPA1 overexpression protected from these multiple and different death stimuli in vivo. In immortalized fibroblasts, OPA1 modulates mitochondrial apoptosis (Frezza et al., 2006) as well as RCS assembly and mitochondrial respiration efficiency (Cogliati et al., 2013). We therefore sought to verify if the same held true in primary tissues. As expected, mitochondria were longer in primary myoblasts prepared from *Opa1*^*tg*^ diaphragms (Figures 6A and 6B), and cristae tighter in hearts (Figures 6C and 6D) as well as in other organs (data not shown) from *Opa1*^*tg*^ mice. Functionally, *Opa1*^*tg*^ primary fibroblasts produced less mitochondrial reactive oxygen species (ROS) when supplied with excess glucose or galactose that forces mitochondrial respiration (Figure 6E). This reduction of ROS production was not caused by desensitization of the mitochondrial permeability transition pore (PTP) that aggravates ROS production and apoptosis (Basso et al., 2005), since complex IV-dependent mitochondrial respiration (Figure S5A), membrane potential (Figure S5B), and basal and maximal calcium retention capacity (CRC; a sensitive indicator of PTP threshold; Figures S5C and S5D) were superimposable in WT and *Opa1*^*tg*^ mitochondria. Conversely, respiratory control ratio (RCR) of *Opa1*^*tg*^ liver and fibroblast mitochondria energized with glutamate/malate is higher (Cogliati et al., 2013), supporting reduced ROS production from stabilized RCSs. In addition to reduced ROS production, *Opa1*^*tg*^ mitochondria were releasing less cytochrome c in an established assay of mitochondrial permeabilization based on recombinant active caspase-cleaved BID (cBID) (Scorrano et al., 2002; Frezza et al., 2006) (Figure 6F), notwithstanding that BAK activation (measured as its oligomerization) in response to cBID was comparable in WT and *Opa1*^*tg*^ mitochondria (Figure S5E). To verify if cytochrome c mobilization from the cristae was blunted in *Opa1*^*tg*^ mitochondria we measured the ratio of ascorbate (asc)-driven over tetramethyl-p-phenylenediamine (TMPD)-driven respiration, an established assay of submitochondrial cytochrome c localization (Scorrano et al., 2002). While basal asc/TMPD ratios were comparable, cBID increased it only in WT mitochondria (Figure 6G), indicating that the apoptotic redistribution of cytochrome c was blocked in *Opa1*^*tg*^ mitochondria. Accordingly, the OPA1 oligomers targeted by cBID to trigger cristae remodeling and cytochrome c redistribution (Frezza et al., 2006) were stabilized in *Opa1*^*tg*^ mitochondria (Figures 6H, 6I, and S5E). Taken together these results indicate that mild OPA1 overexpression blunts apoptotic cristae remodeling in vivo.

## Discussion

The field of mitochondrial dynamics blossomed when the processes of cristae remodeling and mitochondrial fragmentation during apoptosis were discovered (Scorrano et al., 2002; Frank et al., 2001; Martinou et al., 1999). The discovery that OPA1 controls cristae remodeling (Frezza et al., 2006) and RCS assembly and stability (Cogliati et al., 2013) offered a conceptual framework to explore whether OPA1 overexpression interfered with cell death in vivo. Our results indicate that mild OPA1 overexpression is compatible with life and protects mice from multiple form of damage, implicating cristae remodeling in myocardial and brain infarction, hepatocellular apoptosis, and muscular atrophy.

In order to investigate the role of cristae remodeling in vivo we elected to modulate levels of its master regulator OPA1. Not surprisingly, given the crucial role of this protein in mitochondrial physiology, its ablation is lethal early during intrauterine development (Davies et al., 2007), and its inducible deletion in differentiated tissues such as brain and skeletal muscle is similarly fatal (data not shown). We therefore turned to a different approach and generated a mouse model of targeted, controlled *Opa1* overexpression (Cogliati et al., 2013) where we could avoid the paradoxical toxic effects of high OPA1 levels (Cipolat et al., 2004).

*Opa1* overexpression does not impair fertility, intrauterine development, adult life, and lifespan, a surprising lack of effect for a gene with a documented anti-apoptotic effect. Conversely, in the BL6 background it slightly, but significantly, lowered body weight at 5–9 months, and in both BL6 and Sv129 backgrounds it granted protection from a panoply of insults to different tissues. It shall be mentioned, however, that in the Sv129 background the incidence of spontaneous cancer is increased, and lifespan is accordingly reduced (data not shown), unlike the crucial apoptosis inhibitors *Bcl*-2 and *Bcl*-X_L_ whose overexpression does not promote cancer per se, except when it is confined in the B cell compartment (Frenzel et al., 2009; Pena et al., 1998). The small effect of *Opa1* overexpression on body weight might be a consequence of its role on mitochondrial bioenergetics, or it could result from a yet-unclear effect of cristae shape on intermediate metabolism. In any case, it suggests that the relationship between cristae shape and weight control shall be addressed experimentally. Finally, the increase in cancer prevalence offers a potential explanation as to why animals do not display constitutively high OPA1 levels that are conversely beneficial against adult life cell death.

Chronic muscle disuse induced by denervation results in muscle atrophy with accompanying mitochondrial alterations (Adhihetty et al., 2007) and network disruption (Romanello et al., 2010). The anti-atrophic effect of OPA1 overexpression was not confined to acute muscle loss caused by denervation, but also counteracted chronic muscle loss and myopathy caused by a genetic mitochondrial defect (Civiletto et al., 2015 [this issue of *Cell Metabolism*]), further substantiating the key role of OPA1 in the control of muscle atrophy. Mechanistically, OPA1 overexpression did not stimulate mitochondrial biogenesis or blunted autophagy, but it sustained mitochondrial function. OPA1 expression indeed triggered a slight mitochondrial elongation, which was accompanied by a remarkable increase in respiratory capacity that correlated with cristae tightness (Cogliati et al., 2013). Thus, maintenance of mitochondrial cristae seems a feasible strategy to counteract muscular atrophy, an emerging medical problem in the aging society.

In addition to skeletal muscle, brain and heart are also high-energy-demanding tissues where mitochondrial dysfunction is detrimental. Indeed, ischemia and I/R injury are associated with a dramatic change in mitochondrial morphology and ultrastructure (Calo et al., 2013). In addition to its role in apoptosis, mitochondrial dynamics has recently been implied in necrosis and necroptosis (Orogo and Gustafsson, 2013; Wang et al., 2012). Mild OPA1 overexpression was sufficient to improve enzymatic parameters associated with tissue damage in response to ischemic injury. Our results suggest that cristae remodeling is central also to propagate necrotic damage, and that its prevention can ameliorate the ischemic damage outcome. Notably, OPA1 overexpression did not increase brain or heart vascularization (data not shown), suggesting a direct effect on the mechanisms of ischemic cell damage. During muscle atrophy as well as heart I/R, long OPA1 forms appear to be cleaved, presumably by the stress-activated ATP-independent metalloprotease OMA1 (Baker et al., 2014), to inactivate OPA1 function that depends in full (Anand et al., 2014) or at least partially (Cipolat et al., 2006) on its long membrane inserted forms. Since stabilization of long OPA1 in *Opa1*^*tg*^ mice was associated with I/R and muscular atrophy protection, it is tempting to speculate that OPA1 cleavage inhibitors could be cardio and muscle protective. Finally, the importance of OPA1 in brain and muscle is further corroborated by the finding that its overexpression ameliorates two mouse models of mitochondrial diseases characterized by profound neurological and muscular defects (Civiletto et al., 2015 [this issue of *Cell Metabolism*]).

*Opa1*^*tg*^ hearts at 9 months are hypertrophic. Our analysis failed to reveal any histological or functional sign of maladaptive hypertrophy or of increased afterload, one of the most common causes of hypertrophy. We can speculate that *Opa1* overexpression results in physiological hypertrophy because it blunts normal atrophic processes also in the heart, or that the improved *Opa1*^*tg*^ mouse motor performance results in an endurance training type of hypertrophy, or that the ameliorated *Opa1*^*tg*^ mitochondrial function directly results in increased myocardial mass, directly linking mitochondrial function to physiological hypertrophy. Irrespective of its underlying mechanism, the observed hypertrophy does not account for *Opa1*^*tg*^ heart protection from I/R, measured in isolated preparations and before its onset.

The liver, where Fas is constitutively expressed on hepatocytes (Ogasawara et al., 1993), is very sensitive to Fas-mediated apoptosis. Because hepatocytes are type II cells in which Fas-induced death is dependent on the mitochondrial pathway, Fas receptor activation in the liver has been widely employed to study apoptosis in vivo (Rodriguez et al., 1996; Yin et al., 1999; Wei et al., 2001). Notably, OPA1 overexpression also protected from Fas-mediated hepatocellular apoptosis. While OPA1 expression did not interfere with Bax, Bak mediated outer membrane permeabilization, and it blunted cytochrome c release in vitro and in vivo by counteracting its intramitochondrial redistribution during cristae remodeling.

Our work unravels a role for OPA1 and mitochondrial cristae remodeling in multiple forms of cell death in vivo. The analysis of a mouse model of mild, targeted OPA1 overexpression extends the types of cell death controlled by cristae remodeling beyond apoptosis, indicates a crucial role for mitochondrial ultrastructure in tissue adaptation to pathological stimuli, and offers a proof of principle for therapies aimed at ameliorating mitochondrial cristae in acute and chronic diseases.

## Experimental Procedures

### Generation of *Opa1*^*tg*^ Mice

Experimental procedures for the generation of *Opa1*^*tg*^ mice were described (Cogliati et al., 2013). *Opa1*^*tg*^ mice were initially developed in a mixed genetic background of C57BL/6 × Sv129Ola and were backcrossed to C57BL/6 and Sv129 for more than ten generations. The genotype was determined by analysis of DNA extracted from tail biopsies via PCR. All procedures were authorized by the Office Veterinaire Cantonal of Geneva (1034/3703/2 to L.S.) and the CEASA of the University of Padova (32/2011 to L.S.).

### Echocardiography

Echocardiography was performed in adult (5 and 9 months) *Opa1*^*tg*^ (n = 6) and age- and sex-matched littermate controls (n = 6) using a Vevo 2100 (VisualSonics, Toronto) system equipped with a 30-MHz transducer. Anesthesia was induced with 3% isoflurane, maintained with 1.5% isoflurane during constant monitoring of temperature, respiration rate, and ECG. Details on echocardiography analysis can be found in Supplemental Experimental Procedures.

### Muscle Denervation

Denervation was performed in 5-month-old males by cutting the left hind limb sciatic nerve, with the right hind limb being used as control. At 10 days after denervation animals were sacrificed by cervical dislocation, and muscles were utilized for histological experiments or gene expression studies.

### Heart I/R

Heart I/R was performed using a nonrecirculating Langendorff model (Barbato et al., 1996). The hearts were rapidly excised from cervical-dislocated 5 months sex-matched animals and perfused retrogradely for 10 min with a bicarbonate buffer (118.5 mM NaCl, 3.1 mM KCl, 1.18 mM KH_2_PO_4_, 25.0 mM NaHCO_3_, 1 mM MgCl_2_, 1.4 mM CaCl_2_, and 5.6 mM glucose) insufflated with 37°C 95% O_2_-5% CO_2_ (pH 7.4) at a constant flux of 5 ml/min. After 40 min of ischemia, hearts were reperfused for 15 min. Coronary effluent during the reperfusion period was collected to measure LDH. At the end of reperfusion the hearts were quickly immersed into PBS supplemented with 0.5% Triton X-100 and homogenized for total LDH measurement.

### Brain Ischemia

Animals were sacrificed 3 days after middle cerebral artery (MCA) occlusion. MCA ligation surgery in mice and 2,3,5-Triphenyltetrazolium chloride (TTC) staining for quantification of infarct volumes were performed as described (Colak et al., 2011).

### Fas-Induced Apoptosis

Male littermates 3 months old of the indicated genotypes were tail vein injected with an activating anti-Fas antibody (JO2, 0.25 μg/g body weight in sterile saline solution, BD Pharmingen) or with sterile saline solution as a control. The animals were monitored and sacrificed 24 hr after injection for liver biochemistry or histology and clinical chemistry. Blood samples were collected in BD Microtainer PST LH Tubes and centrifuged at 6,000 g for 10 min to separate plasma. Plasma levels of alanine aminotransferase (ALT) and aspartate aminotransferase (AST) were determined using standard, Clinical Pathology Accredited and ISO9001:2008-certified procedures at the Department of Laboratory Medicine, Padova University Hospital. Liver samples were immediately frozen in liquid nitrogen for biochemical analysis or processed to paraformaldehyde (PFA) fixation for subsequent histological analysis and TUNEL staining.

### Histology and Immunofluorescence Staining

Heart, muscle, and liver tissues were fixed in 1% PFA in PBS at room temperature for 15 min, equilibrated in a sucrose gradient, frozen in liquid nitrogen, sectioned, and processed for histological and immunofluorescence analyses. Sections (10 microns) were obtained with a cryostat (Leica CM1850, Leica Microsystems, Wetzlar) and incubated with primary antibodies diluted in PBS supplemented with 1% BSA and 0.5% Triton X-100 overnight at 4°C. Details on the antibodies used can be found in Supplemental Experimental Procedures. Livers were also cut into small pieces and fixed overnight at 4°C in 4% PFA in PBS and then dehydrated through serial ethanol concentrations for paraffin embedding. Paraffin-embedded livers were used for TUNEL and H&E staining. TUNEL assay was performed by using the ApopTag peroxidase in situ apoptosis detection system, following the procedure suggested by the manufacturer (Chemicon). For COX and SDH histochemical analysis, tissues were frozen in liquid nitrogen pre-cooled isopentane, and 8-μm-thick sections were stained as described (Sciacco and Bonilla, 1996).

### Biochemical Analysis of MRC Complexes

Skeletal and cardiac muscle samples were snap-frozen in liquid nitrogen and homogenized in 10 mM phosphate buffer (pH 7.4). The spectrophotometric activities of CI, CII, CIII, and CIV, as well as citrate synthase (CS), were measured as described (Bugiani et al., 2004).

### Gene Expression Analyses

Total RNA was prepared from gastrocnemius muscles using TRIzol (Invitrogen). cDNA was generated from 0.4 μg of RNA reverse-transcribed with SuperScript III Reverse Transcriptase (Invitrogen). Duplicates of cDNA samples were then amplified on the 7900HT Fast Real-Time PCR System (Applied Biosystems) using the Power SYBR Green RT-PCR kit (Applied Biosystems). All data were normalized to GAPDH expression and plotted in arbitrary units as mean ± SEM of independent experiments. The oligonucleotide primers used can be found in Supplemental Experimental Procedures.

### Assays of Mitochondrial Membrane Potential

Mitochondrial membrane potential in isolated flexor digitorum brevis (FDB) muscle fibers was measured by intensity of TMRM fluorescence. Details can be found in Supplemental Experimental Procedures.

### In Vitro Mitochondrial Assays

Liver and muscle mitochondria were isolated as described (Frezza et al., 2007). Cytochrome c redistribution and release in response to 40 pmol cBID/mg mitochondria was performed as described (Scorrano et al., 2002). Mitochondrial oxygen consumption was measured with a Clark type oxygen electrode (Hansatech Instruments) as described (Frezza et al., 2007). Details on mitochondria Ca^2+^ retention capacity and mitochondrial membrane potential measurements can be found in Supplemental Experimental Procedures.

### Biochemistry

For protein crosslinking, mitochondria were treated as indicated with 1 mM EDC or 1 mM BMH as described (Frezza et al., 2006). For SDS-PAGE, equal amounts of mitochondrial proteins were separated on 3%–8% Tris-acetate or 4%–12% Tris-MES (NuPAGE, Invitrogen) polyacrilamide gel, transferred onto PVDF membranes (Bio-Rad), and probed using the indicated primary antibodies and isotype-matched secondary antibodies conjugated to horseradish peroxidase. Signal was detected with ECL (Amersham). Details on the antibodies used can be found in Supplemental Experimental Procedures. Densitometry was performed using ImageJ.

To detect OPA1 oligomers by BN-PAGE, mitochondria were resuspended in an appropriated volume of Buffer D (1 M 6-aminohexanoic acid, 1.25% V/V digitonin, 50 mM Bis-Tris-HCl [pH 7]) at a final concentration of 10 mg/ml. Following centrifugation, the supernatant was collected, and 5% Serva Blue G dye in 1 M 6-aminohexanoic acid was added to one-third of the final volume of the sample. Equal amounts (100 μg) of mitochondrial proteins were separated on a 3%–12% gradient BNGE (Invitrogen) as described (Schägger, 1995).

### Determination of ROS Production

Mitochondrial ROS levels were analyzed in cells incubated for 24 hr in DMEM containing glucose (4.5 mg/ml) or galactose (0.9 mg/ml). Cells were stained with 2 μM MitoSOX (Invitrogen) in HBSS for 30 min at 37°C and rinsed, and the fluorescence was assessed by flow cytometry using the FL1 channel of a FACSCalibur (BD Pharmingen) cytometer.

### Transmission EM

Heart specimens from mice of the indicated genotype were fixed in 2% formaldehyde, 2.5% (V/V) glutaraldehyde in 0.1 M Na-cacodylate (pH 7.4) for 2 hr at room temperature and then overnight at 4°C. EM was performed as described (Scorrano et al., 2002). Thin sections were imaged on a Tecnai-20 electron microscope (Philips-FEI).

## Author Contributions

T.V. performed experiments, analyzed data, and wrote the manuscript; M.E.S. generated the Opa1^tg^ colony and performed experiments; V.R. analyzed denervated muscles; T.Z. analyzed hearts; R.Q.-C. performed ROS measurements; M. Semenzato performed RT-PCR analyses; R.M. helped perform heart ischemia reperfusion; V.C. performed mitochondrial in vitro assays; G.C. performed respiratory chain activity staining; P.P. helped perform echocardiography; C.V., M.Z., F.D.L., M.M., and M. Sandri supervised research, analyzed data, and contributed to writing; and L.S. conceived the project, supervised research, analyzed data, and wrote the manuscript.

## Supplemental Information

Document S1. Figures S1–S5 and Supplemental Experimental Procedures

Document S2. Article plus Supplemental Information
