# Supplementary material for: The Opa1-Dependent Mitochondrial Cristae Remodeling Pathway Controls Atrophic, Apoptotic, and Ischemic Tissue Damage
Source: Cell Metab. 2015 Jun 2;21(6):834–44. doi: 10.1016/j.cmet.2015.05.007 (PMC4457892; doi:10.1016/j.cmet.2015.05.007)
Supplement: Document S1. Figures S1–S5 and Supplemental Experimental Procedures [file mmc1.pdf]

Cell Metabolism
Supplemental Information

The Opa1-Dependent Mitochondrial Cristae Remodeling Pathway Controls Atrophic, Apoptotic, and Ischemic Tissue Damage

Tatiana Varanita, Maria Eugenia Soriano, Vanina Romanello, Tania Zaglia, Rubén Quintana-Cabrera, Martina Semenzato, Roberta Menabò, Veronica Costa, Gabriele Civiletto, Paola Pesce, Carlo Viscomi, Massimo Zeviani, Fabio Di Lisa, Marco Mongillo, Marco Sandri, and Luca Scorrano

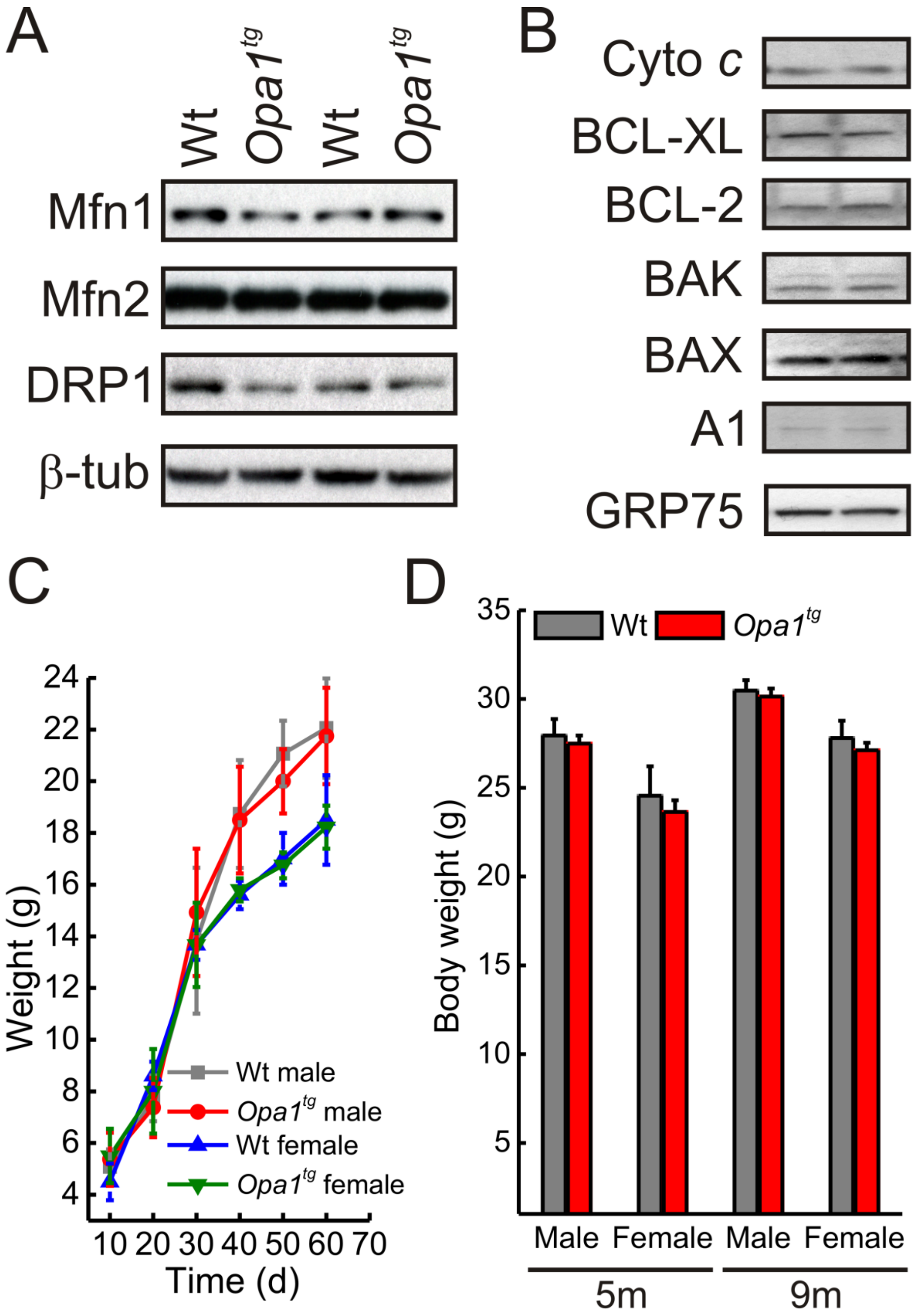

Supplemental Figure 1. Levels of mitochondria-shaping and apoptosis proteins are not changed in *Opa1*^{tg} Mice, Related to Fig. 1

(A) Equal amounts of protein from liver tissue of the indicated genotypes were separated by SDS-PAGE and immunoblotted with the indicated antibodies.

(B) Equal amounts of protein from liver tissue of the indicated genotypes were separated by SDS-PAGE and immunoblotted with the indicated antibodies.

(C) Body weight of C57/Bl6 individuals of the indicated genotype and sex for the indicated time. Data represent average + SEM (n=20 per each group).

(D) Body weight is represented as average + SEM of 5 month males (n=19 for each group) and females (n= 12 for each group) and 9 month old males (n= 32 for each group) and females (n=18 for each group) Sv129 littermates of the indicated genotype.

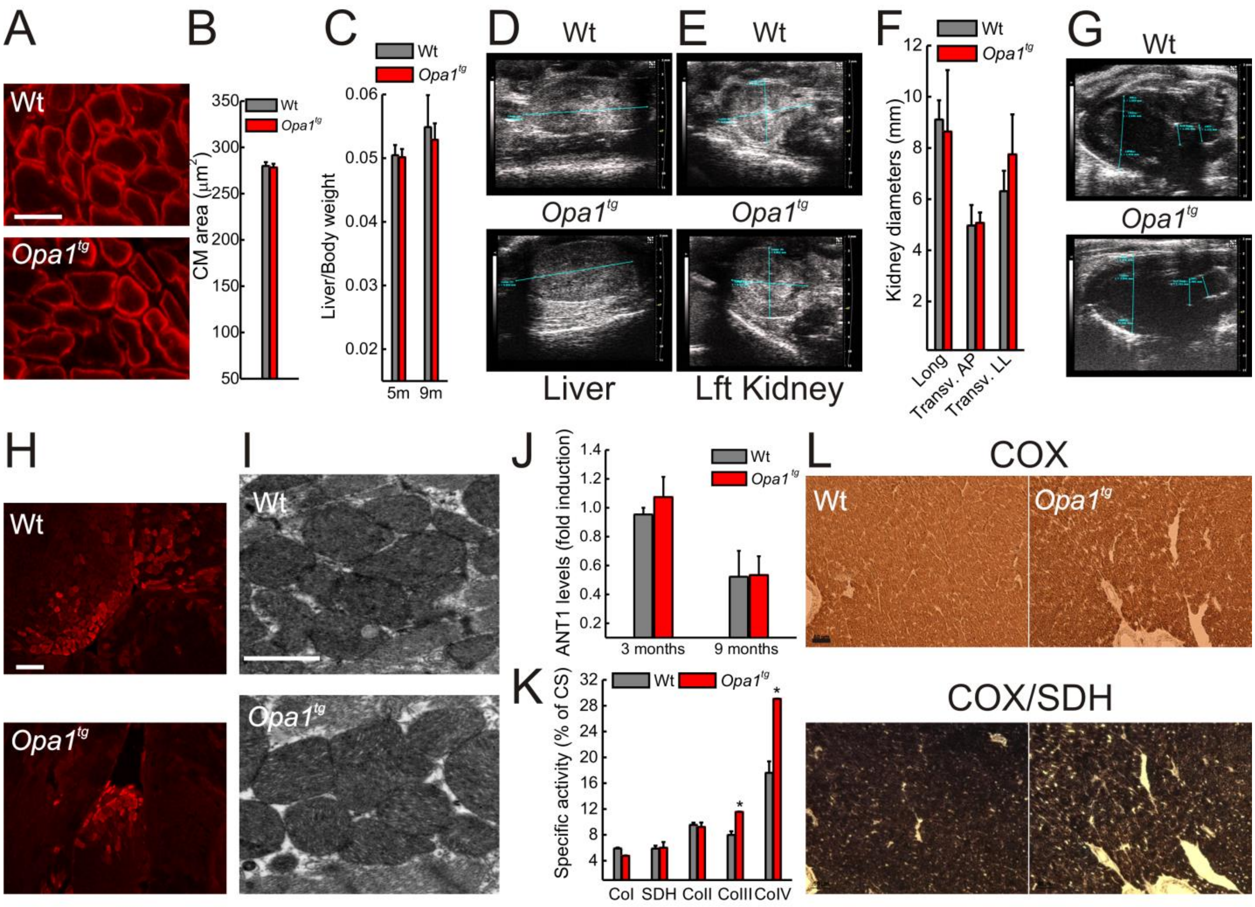

Supplemental Figure 2. Histological and Morphological Characterization of *Opa1*^{tg} Heart, Liver and Kidney, Related to Fig. 2

(A) Immunofluorescence analysis on ventricular cryosections from mice of the indicated genotype stained with an antibody to dystrophin. Images are details from the left ventricle. Scale bar, 25 µm.

(B) Quantification of cardiomyocyte (CM) area in cryosections from 5 month old hearts of the indicated genotype in experiments as in (A). Data are mean±SD of 5 individuals for each group).

(C) Quantification of liver weight/body weight ratio in 5 and 9 month old male mice of the indicated genotypes. Data represent average + SEM (n=10 for each group).

(D) Echography images of livers from 9 month old mice of the indicated genotype.

(E) Echography short axis view of the left kidney from 9 month old mice of the indicated genotype.

(F) Morphometric analysis of kidney from 9 month old mice of the indicated genotype in experiments as in (E). Data are mean±SD of 6 individuals in each group)

(G) Echocardiographic long axis view of hearts from 5 month old littermates of the indicated genotype. LV: left ventricle; A: aorta.

(H) Representative confocal images of ventricular cryosections from mice stained for beta-myosin heavy chain (β-MHC). Scale bar, 100 µm.

(I) Electron micrographs of hearts from 9 month old littermates of the indicated genotyopes. Scale bar, 1µm.

(J) Data represent average±SEM of the ANT1 mRNA levels determined by RT-PCR in hearts from littermates of the indicated genotype of the indicated age (n=3 for each group).

(K) Mitochondrial respiratory chain specific activities measured in hearts of the indicated genotypes normalized to that of citrate synthase. Data are average±SEM of 3 individuals for each group. *, $p<0.05$ in an unpaired two-sample Student's t-tests.

(L). Representative images of COX and double COX/SDH histological staining in heart sections from 9 month old littermates of the indicated genotypes. Scale bar, 50 µm.

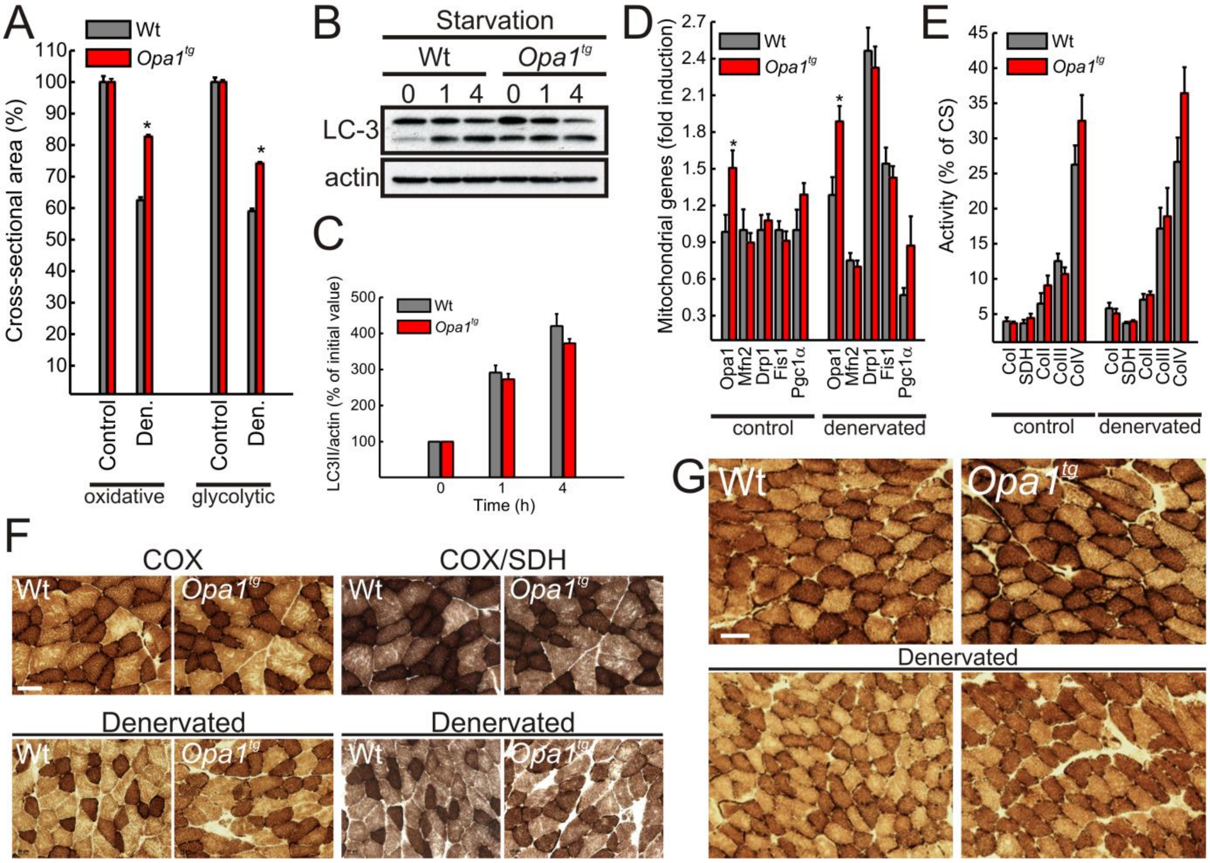

Supplemental Figure 3. Opa1^{tg} mice are protected from denervation induced muscular atrophy, related to Figure 3

(A) Cross-sectional area (CSA) measurements of innervated and denervated fibers of the indicated type 10 days after denervation. Data are average±SEM of 5 independent experiments. *, p<0.05 in an unpaired two-sample Student's t-tests.

(B) Autophagy was induced in mouse adult fibroblasts (MAFs) of the indicated genotypes by starvation in the presence of 100µM chloroquine, cells were lysed at the indicated time points and equal amounts of proteins were separated by SDS-PAGE and immunoblotted using the indicated antibodies.

(C) Densitometric analysis of experiments as in (B). Data represent average±SEM of 5 independent experiments and are normalized to the initial value.

(D) Data represent average±SEM of the indicated mRNA levels determined by RT-PCR in littermates of the indicated genotype treated as indicated (n=5 for each group). *, p<0.05 in an unpaired two-sample Student's t-tests.

(E) Mitochondrial respiratory chain specific activities measured in muscles of the indicated genotypes treated as indicated normalized to that of citrate synthase. Data are average±SEM of 5 individuals for each group. *, p<0.05 in an unpaired two-sample Student's t-tests.

(F, G) Representative images of COX and COX/SDH histological staining in control and denervated gastrocnemius (F) and soleus (G) muscles from littermates of the indicated genotypes treated as indicated. Scale bars, 50µm.

A

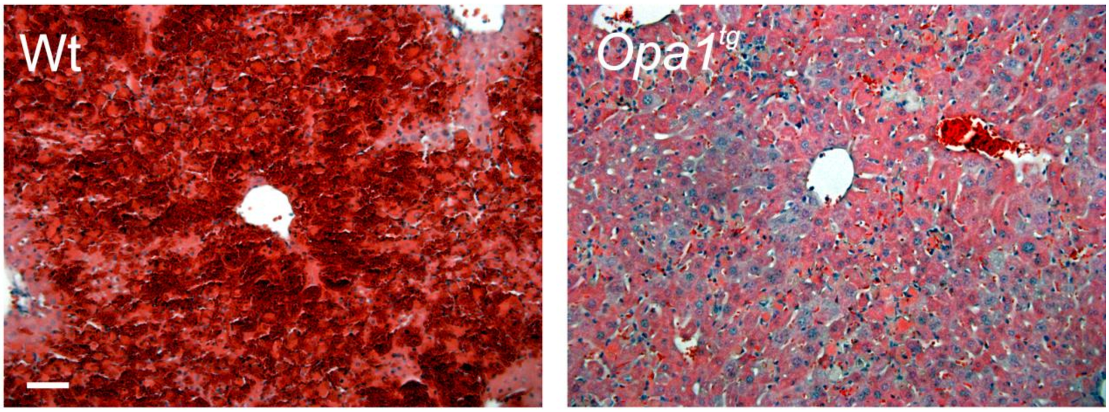

B

Genotype	Dead/injected
Wt	3/4
Opa1^{tg}	2/4

Supplemental Figure 4. Sv129 *Opa1^{tg}* Livers are ProtectedF rom Fas-Induced Damage, Related to Fig. 5

(A) Representative images of Hematoxylin-Eosin stained paraffin embedded liver sections from littermates of the indicated genotype tail vein injected with 0.25µg/g anti-Fas antibody (Fas) and sacrificed after 24h. Scale bar, 50 µm.

(B) Number of dead Sv129 mice of the indicated genotype 24 h after tail vein injection of 0.25µg/g anti-Fas antibody

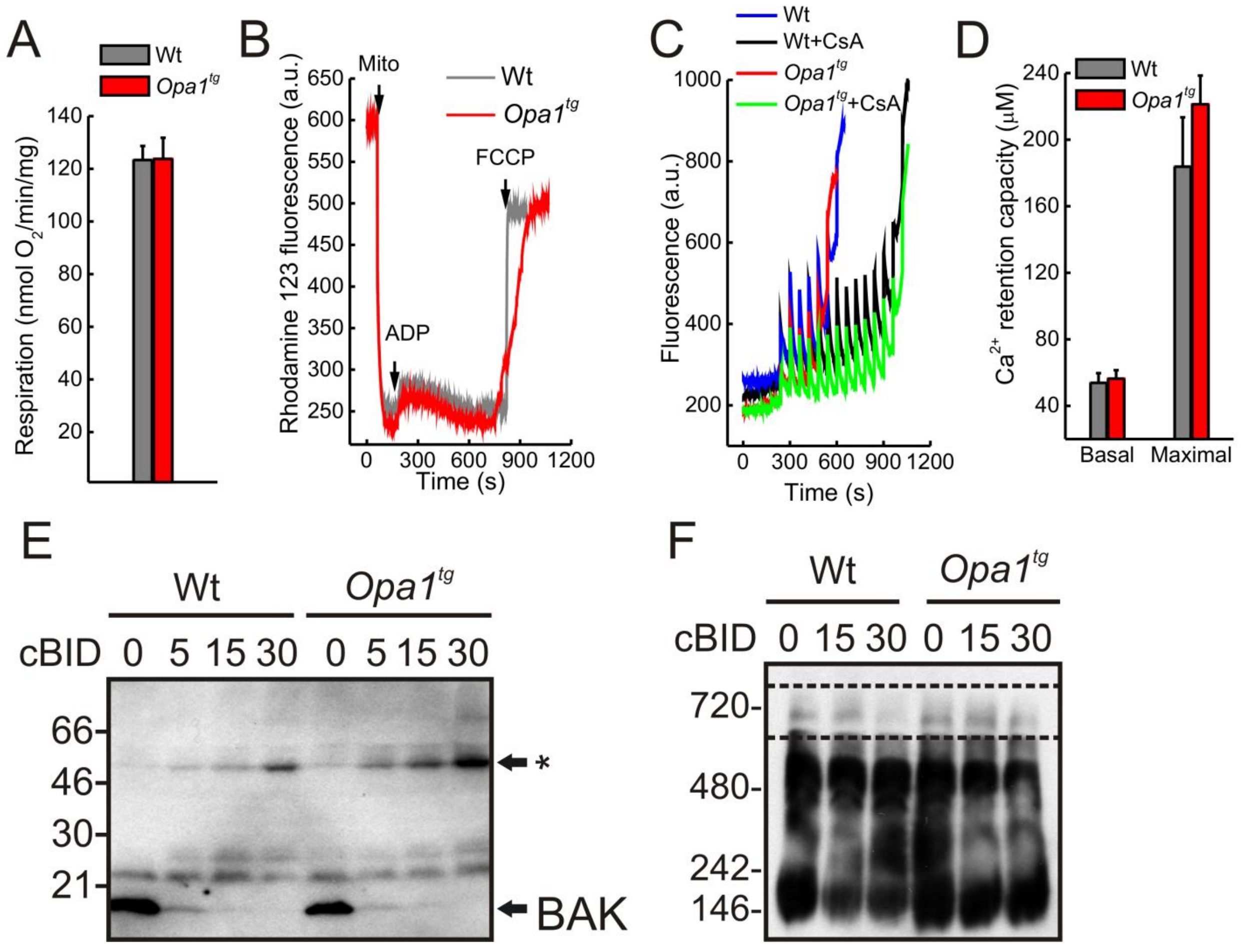

Supplemental Figure 5. *Opa1^{tg}* Mitochondrial Function Is Not Altered, Related to Figure 6

(A) Complex IV dependent respiration of mitochondria isolated from livers of the indicated genotypes. Data represent average $\pm$ SEM (n=4 for each group).

(B) Representative traces of Rhodamine 123 fluorescence. Mitochondria isolated from livers of the indicated genotype (1 mg/mL, Mito) were treated where indicated (arrows) with 300 μM ADP and 200 nM FCCP.

(C) Representative traces of Calcium Retention Capacity (CRC) of liver mitochondria isolated from 5 month old mice of the indicated genotype. For the assessment of the maximal CRC, 2 μM Cyclosporine A (CsA) was present in the medium.

(D) Quantification of CRC in experiments as in (C).

(E) Mitochondria of the indicated genotype were treated with cBID for the indicated times, crosslinked with. 10 mM BMH and after 30 min the crosslinking reaction was quenched. Equal amounts (40μg) of proteins were analyzed by SDS-PAGE/immunoblotting using anti-BAK antibody. Asterisks:BAK multimers.

(F) Representative BNGE analysis of OPA1 oligomers in muscle mitochondria of the indicated genotypes treated for the indicated times with cBID. The boxed area indicates the OPA1 high molecular weight complexes.

Supplemental Experimental Procedures

Ecocardiography

Two-dimensional cine loops with frame rates of 200 frames/s of a long-axis view and a short-axis view at proximal-, mid- and apical level of the left ventricle (LV) were recorded. Inter ventricular septum (IVS) and LV posterior wall (LVPW) thicknesses, LV internal diameter (LVID) and maximal LV length were measured in systole (s) and in diastole (d) from the long axis B-mode image, according to standard procedures. Ejection fraction (EF) was determined by the following formula: Simp EF (%) = 100 x Simp Systolic Volume (SV)/ Simp LV volume; d. To assess changes in LV shape, the sphericity index (SI) was calculated at end-diastole (d) and end-systole (s) as the volume of the LV divided by the volume of a sphere with a diameter equal to the LV longest axis (measured in the apical view) using the following formulas: SI: d=LV volume; $d/(3/4*\Pi*(\text{Simp Length; d})^3)$; SI: s = LV volume; $s/(3/4*\Pi*(\text{ Simp Length; s})^3)$.

As this ratio increases, the ventricle becomes more spherical. Echocardiographic image acquisition and analysis were performed by a single operator, blinded to the mouse genotype.

Gene expression analyses

The following oligonucleotides primers were employed:

Opa1 forward 5’-ATACTGGGATCTGCTGTTGG-3’ reverse 5’-AAGTCAGGCACAATCCACTT; *Drp1* forward 5’-TCAGATCGTCGTAGTGGGAA reverse 5’-TCTTCTGGTGAAACGTGGAC-3’; *Fis1* forward 5’-AAGTATGTGCGAGGGCTGT-3’ reverse 5’-TGCCTACCAGTCCATCTTTC-3’; *Mfn2* forward 5’-ATGTTACCACGGAGCTGGAC-3’ reverse 5’-AACTGCTTCTCCGTCTGCAT-3’; *Pgc1a1* forward 5’-GGAATGCACCGTAAATCTGC-3’ reverse 5’-TTCTCAAGAGCAGCGAAAGC-3’; *Atrogin-1* forward 5’-GCAAACACTGCCACATTCTCTC-3’ reverse 5’-CTTGAGGGGAAAGTGAGACG-3’; *MuRF-1* forward 5’-

ACCTGCTGGTGGAAAACATC-3' reverse 5'-ACCTGCTGGTGGAAAACATC-3'; *Musa1* forward 5'-TCGTGGAATGGTAATCTTGC-3' reverse 5'-CCTCCCGTTTCTCTATCACG-3'; *Bnip3* forward 5'-TTCCACTAGCACCTTCTGATGA-3' reverse 5'-GAACACCGCATTTACAGAACAA-3'; *CathepsinL* forward 5'-GTGGACTGTTCTCACGCTCAAG-3' reverse 5'-TCCGTCCTTCGCTTCATAGG-3'; *FoxO1* forward 5'-GCTGGGTGTCAGGCTAAGAG-3' reverse 5'-GAGGGGTGAAGGGCATCT-3'; *FoxO3* forward 5'-CGCTGTGTGCCCTACTTCA-3' reverse 5'-CCCGTGCCTTCATTCTGA-3'; *FoxO4* forward 5'-CGGAGTGAAAGGGACAGTTTAG-3' reverse 5'-CCCTGTGGCTGACTTCTTATTC-3'; *LC3b* forward 5'-CACTGCTCTGTCTTGTGTAGGTTG-3' reverse 5'-TCGTTGTGCCTTTATTAGTGCATC-3'; *p62* forward 5'-CCCAGTGTCTTGGCATTCTT-3' reverse 5'-AGGGAAAGCAGAGGAAGCTC-3'; *ActR2B* forward 5'-TTAAGGATCACTGGCTGAAACA-3' reverse 5'-GGATACCCGCTCTTCTACACAG-3'; *Myostatin* forward 5'-AGTAAAAGCCCAACTGTGGATA-3' reverse 5'-TCATGTCAAGTTTCAGAGATCG-3'; *Alk3* forward 5'-CTGGGAGTGGATCTGGATTG-3' reverse 5'-CTGTTTGGCAATAGTTCGCTG-3'; *Gapdh* forward 5'-CACCATCTTCCAGGAGCGAG-3' reverse 5'-CCTTCTCCATGGTGGTGAAGAC-3'; *Mul1* forward 5'- AGGGCATTCTTTCAGAAGCA-3' reverse 5'-GGGGTGGAACTTCTCGTACA-3'; *Actin* 5'-forward 5'-CTGGCTCCTAGCACCATGAAGAT-3' reverse 5'-GGTGGACAGTGAGGCCAGGAT-3'; *Anp* forward 5'-GCAGAGACAGCAAACATCAGA-3' reverse 5'-GCATCTTCTCCTCCAGGTG-3'.

Biochemistry

The following primary antibodies were used: Monoclonal anti-OPA1 (1:1000 BD), rabbit polyclonal anti-MFN1 (1:1000 Millipore), monoclonal anti-MFN2 (1:1000 Abnova), monoclonal anti-DLP1 (1:1000 BD), monoclonal anti-Cytochrome c (1:1000 BD Pharmingen), rabbit polyclonal anti-BCL-XL (1:1000 Santa Cruz Biotechnology) , rabbit polyclonal anti-BCL-2 (1:1000 Santa Cruz Biotechnology), rabbit polyclonal anti-BAK-NT (1:1000 Upstate), rabbit polyclonal

anti-BAX (1:1000 Millipore), rabbit polyclonal anti-GRP75 (1:1000 Santa Cruz Biotechnology), rabbit polyclonal anti-A1 (1:1000 Santa Cruz Biotechnology), monoclonal anti-ACTIN (1:1000 Chemicon), monoclonal anti-CII (1:2000 Abcam).

Autophagy analysis

Autophagy was induced by serum starvation. Cells of the indicated genotypes were washed 4 times and then incubated in Earle's Balanced Salt Solution (EBSS) at 37°C for the indicated time. To block autophagic flux, 100 μM chloroquine (CQ) was added.

Immunohistochemistry

We used the following antibodies: rabbit anti-dystrophin (1:600 Abcam), rabbit anti-collagen I (1:800 Acris), rabbit anti-Atrial Natriuretic Peptide (ANP, 1:500 Peninsula Lab), monoclonal anti-cytochrome c (1:200 BD Pharmingen), rabbit polyclonal anti-TOM20 (1:200 Santa Cruz Biotechnology). To detect the primary antibodies the following secondary antibodies were used: Alexa Fluor 488, or 568 (1:200 Invitrogen), FITC and TRITC-conjugated secondary antibodies (Jackson Labs).

Assays of mitochondrial membrane potential

FDB myofibres were placed in 1ml Tyrode's buffer and loaded with 5 nM TMRM (Molecular Probes) supplemented with 1 μM cyclosporine H (a P-glycoprotein inhibitor) for 30 min at 37°C. Myofibres were then observed using an Olympus IX81 inverted microscope (Melville, NY) equipped with a CellR imaging system. Sequential images of TMRM fluorescence were acquired

every 60s with a 4X objective (Olympus). Where indicated, oligomycin (5nM, Sigma) or the uncoupler carbonyl cyanide p-trifluoromethoxyphenylhydrazone (FCCP, 400nM, Sigma) was added. TMRM fluorescence analysis over the mitochondrial regions of interest was performed using ImageJ.

In vitro mitochondrial assays

Mitochondria Ca^{2+} retention capacity was measured fluorimetrically using a Perkin Elmer LS50B fluorimeter (λ_{ex} 505 nm; λ_{em}: 535 nm). Freshly isolated mitochondria were resuspended in experimental buffer (EB) (Frezza et al., 2007) supplemented with 5mM glutamate/2.5mM malate and 1µM Calcium Green-5N (Invitrogen). Pulses of 10µM Ca^{2+} were added every 30 seconds until PTP induction. For the maximal CRC, EB was supplemented with 2µM Cyclosporine A.

To measure mitochondrial membrane potential EB was supplemented with 0.2µM Rhodamine 123 and fluorescence was measured using a Perkin Elmer LS50B fluorimeter (λ_{ex} 480 nm; λ_{em}: 540 nm)

Morphometric analysis

Cardiomyocyte cross-sectional area was quantified in five non-consecutive cryosections from the mid-portion of the ventricles per heart were stained with an antibody specific for dystrophin and analyzed by fluorescence microscopy. The images of twelve randomly chosen fields of 69000 μm^2 of area from the ventricles were acquired with a fluorescence microscope (Leica DC130, Leica Microscopes, Germany) and cardiomyocyte cross-sectional area was evaluated using the software ImageJ.

Fiber Cross-Sectional Area (CSA) was measured using ImageJ. More than 1000 fibers per each muscle were counted. The fields were randomly selected to measure the fiber area and all the fibers encompassed in those fields were evaluated. This analysis was carried out in a blind fashion, with the operator unaware of the mice genotype.

Mitochondrial length was measured using the ImageJ Plugin *Mosaic Squassh* (Rizk et al., 2014)

Imaging

Hematoxylin and eosin (H&E), COX/SDH and TUNEL were performed on paraffin embedded sections or cryosections, immunofluorescence was performed on cryosections as previously described. H&E, COX/SDH and TUNEL were imaged with a bright field on fluorescence microscope (Leica DM 5000B) using 20x or 40x PlanApo objectives (Leica). Confocal images were acquired using a confocal microscope (TCS SP5; Leica) using the LAS AF software (Leica), a 63×, 1.4 NA objective, and the 488-nm and 561-nm laser lines.

Supplemental References

Frezza,C., Cipolat,S., and Scorrano,L. (2007). Organelle isolation: functional mitochondria from mouse liver, muscle and cultured fibroblasts. Nat. Protoc. 2, 287-295.

Rizk,A., Paul,G., Incardona,P., Bugarski,M., Mansouri,M., Niemann,A., Ziegler,U., Berger,P., and Sbalzarini,I.F. (2014). Segmentation and quantification of subcellular structures in fluorescence microscopy images using Squassh. Nature Protocols 9, 586-596.
