# Supplementary material for: The Opa1-Dependent Mitochondrial Cristae Remodeling Pathway Controls Atrophic, Apoptotic, and Ischemic Tissue Damage
Source: Cell Metab. 2015 Jun 2;21(6):834–44. doi: 10.1016/j.cmet.2015.05.007 (PMC4457892; doi:10.1016/j.cmet.2015.05.007)
Supplement: Document S2. Article plus Supplemental Information [file mmc2.pdf]

Cell Metabolism

Article

The Opa1-Dependent Mitochondrial Cristae Remodeling Pathway Controls Atrophic, Apoptotic, and Ischemic Tissue Damage

Graphical Abstract

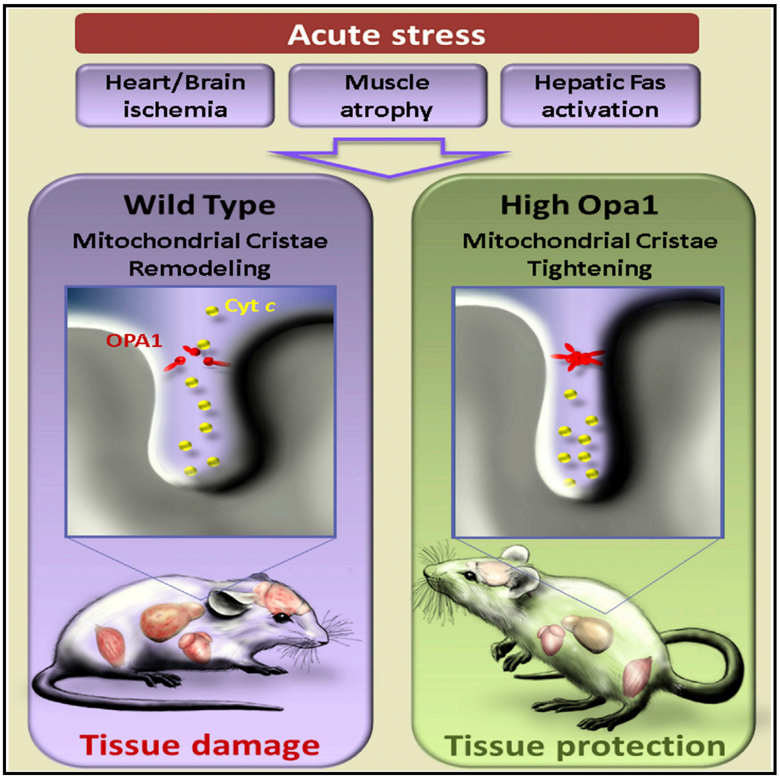

Highlights

- Mice with controlled Opa1 overexpression are viable and grow normally
- Opa1 protects from muscular atrophy, heart and brain ischemia, and liver apoptosis
- Opa1 reduces ROS production and cytochrome c release
- Opa1-controlled cristae remodeling is a targetable component of tissue damage

Authors

Tatiana Varanita,
Maria Eugenia Soriano, ...,
Marco Sandri, Luca Scorrano

Correspondence

luca.scorrano@unipd.it

In Brief

Varanita et al. show that a mouse model of controlled overexpression of the master mitochondrial cristae biogenetic factor Opa1 resists skeletal muscle atrophy, heart and brain ischemic damage, and massive liver apoptosis by blunting mitochondrial dysfunction and cytochrome c release. Together with the accompanying paper by Civiletto et al., the authors offer a proof of principle of the potential for therapies targeting cristae remodeling in sporadic and genetic diseases.

Varanita et al., 2015, Cell Metabolism *21*, 834–844
June 2, 2015 ©2015 The Authors
http://dx.doi.org/10.1016/j.cmet.2015.05.007

Cell Metabolism
Article

The Opa1-Dependent Mitochondrial Cristae Remodeling Pathway Controls Atrophic, Apoptotic, and Ischemic Tissue Damage

Tatiana Varanita,[1,2] Maria Eugenia Soriano,[1,2] Vanina Romanello,[1,3] Tania Zaglia,[3] Rubén Quintana-Cabrera,[1,2] Martina Semenzato,[1,2] Roberta Menabò,[6] Veronica Costa,[1] Gabriele Civiletto,[4,5] Paola Pesce,[7] Carlo Viscomi,[4,5] Massimo Zeviani,[4,5] Fabio Di Lisa,[3] Marco Mongillo,[3] Marco Sandri,[1,3] and Luca Scorrano[1,2,*]
[1]Dulbecco Telethon Institute, Venetian Institute of Molecular Medicine, Via Orus 2, 35129 Padova, Italy
[2]Department of Biology
[3]Department of Biomedical Sciences
University of Padova, Via C. Colombo 3, 35121 Padova, Italy
[4]Fondazione IRCCS Istituto Neurologico ‘‘C. Besta,’’ Via L. Temolo 4, 20126 Milan, Italy
[5]MRC Mitochondrial Biology Unit, MRC Building, Hills Road, Cambridge CB2 0XY, UK
[6]Institute of Neuroscience, National Research Council of Italy (CNR), Via C. Colombo 3, 35121 Padova, Italy
[7]Department of Cardiac, Thoracic and Vascular Sciences, University of Padova, Via Giustiniani, 2, 35128 Padova, Italy
*Correspondence: luca.scorrano@unipd.it
http://dx.doi.org/10.1016/j.cmet.2015.05.007
This is an open access article under the CC BY license (http://creativecommons.org/licenses/by/4.0/).

SUMMARY

Mitochondrial morphological and ultrastructural changes occur during apoptosis and autophagy, but whether they are relevant in vivo for tissue response to damage is unclear. Here we investigate the role of the optic atrophy 1 (OPA1)-dependent cristae remodeling pathway in vivo and provide evidence that it regulates the response of multiple tissues to apoptotic, necrotic, and atrophic stimuli. Genetic inhibition of the cristae remodeling pathway in vivo does not affect development, but protects mice from denervation-induced muscular atrophy, ischemic heart and brain damage, as well as hepatocellular apoptosis. Mechanistically, OPA1-dependent mitochondrial cristae stabilization increases mitochondrial respiratory efficiency and blunts mitochondrial dysfunction, cytochrome c release, and reactive oxygen species production. Our results indicate that the OPA1-dependent cristae remodeling pathway is a fundamental, targetable determinant of tissue damage in vivo.

INTRODUCTION

Mitochondria are crucial organelles in energy conversion (Danial et al., 2003; Rizzuto et al., 2000), and, not surprisingly, impaired mitochondrial function affects organs where energy demand is high, like heart, skeletal muscle, and brain (DiMauro and Schon, 2003). Cellular damage is not only the consequence of mitochondrial bioenergetic failure, given that these organelles also play a crucial role in apoptosis, when in response to several stimuli they release cytochrome c and other pro-apoptotic proteins that execute cell demise (Wang, 2001). Mitochondrial permeabilization during apoptosis is controlled by members of the Bcl-2 family and is accompanied by both morphological and ultrastructural changes of the organelle (Danial and Korsmeyer, 2004; Wasilewski and Scorrano, 2009). Mitochondrial network fragmentation and cristae remodeling with widening of cristae junctions are both required for the complete release of cytochrome c (Scorrano et al., 2002; Yamaguchi et al., 2008).

A family of dynamin-related large GTPases controls mitochondrial fusion and fission (Griparic and van der Bliek, 2001). Fission occurs upon the recruitment of dynamin-related protein 1 (DRP1) on the outer mitochondrial membrane (OMM) (Cereghetti et al., 2008), where it binds to its adaptors, including fission 1 (Fis1), mitochondrial fission factor (MFF), and mitochondrial division (Mid) 49 and 51 (Palmer et al., 2011). Mitochondrial fusion is controlled by mitofusins (MFN) 1 and 2 in the OMM and by OPA1 in the inner mitochondrial membrane (IMM) (Chen et al., 2003; Santel et al., 2003; Cipolat et al., 2004). The IMM can be divided in two subcompartments: the so-called ‘‘boundary membrane’’ and the cristae, separated from the former by narrow tubular junctions (Frey and Mannella, 2000). Upon activation of mitochondrial respiration, cristae transition from the orthodox to the condensed morphology (Hackenbrock, 1966), and during apoptosis they remodel in response to pro-apoptotic BH3-only BCL-2 family members such as BID, BIM-S, and BNIP3, independently from outer membrane permeabilization (Scorrano et al., 2002; Yamaguchi et al., 2008; Landes et al., 2010). In addition to, and independently from, its role in mitochondrial fusion, OPA1 regulates apoptotic cristae remodeling by forming oligomers that participate in cristae junction formation and maintenance (Frezza et al., 2006; Cipolat et al., 2006). Moreover, OPA1, by maintaining mitochondrial cristae morphology, has a direct metabolic effect, stabilizing respiratory chain supercomplexes (RCSs) (Cogliati et al., 2013).

The identification of the key role of OPA1 in cristae remodeling suggests a potential approach to manipulate this process in vivo; however, constitutive as well as conditional tissue-specific *Opa1* ablation in the mouse is lethal (Zhang et al., 2011; Davies et al.,

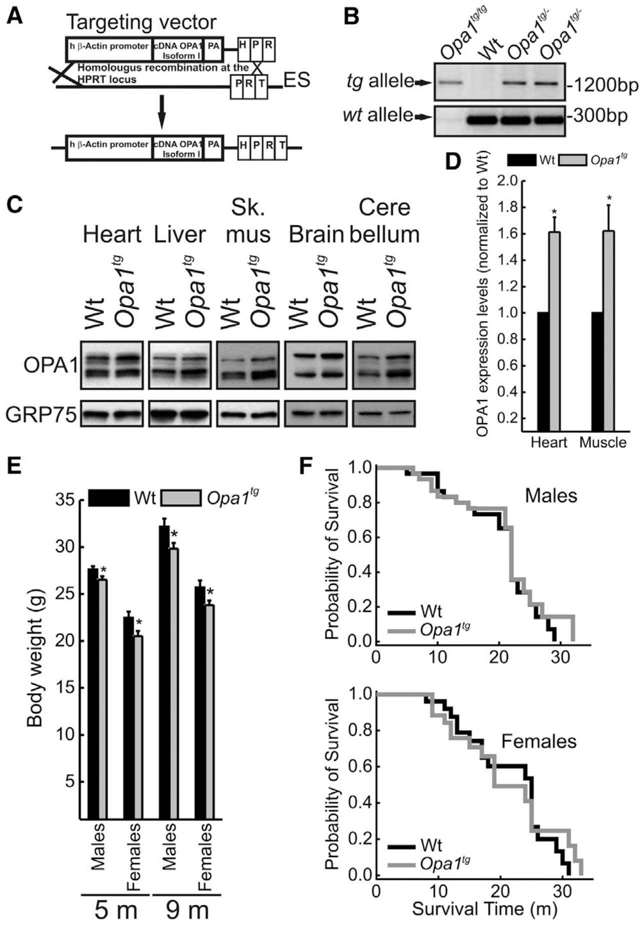

Figure 1. *Opa1^tg* Mice Are Viable, Fertile, and Grow Normally

(A) Schematic representation of targeted transgenesis strategy into the permissive hypoxanthine phosphoribosyltransferase (HPRT) locus. The targeted vector includes a 5′ HPRT homology arm, β-actin promoter, the transgene cDNA OPA1 isoform 1, and 3′ HPRT homology arm including a part of HPRT promoter and a homologous region to the mouse HPRT locus. The targeting construct is linearized before electroporation into BPES cells. Homologous recombination of the 3′ arm reconstitutes a functional HPRT gene, allowing the selection of targeted BPES cells on stringent hypoxanthine-aminopterin-thymidine (HAT) conditions.

(B) PCR analysis of genomic DNA from wild-type (WT), heterozygous (*Opa1*$^{+/-}$), and homozygous (*Opa1*$^{+/+}$) *Opa1^tg* mouse tail.

(C) Equal amounts of protein from tissues of the indicated genotypes were separated by SDS-PAGE and immunoblotted with the indicated antibodies.

(D) Densitometric analysis of OPA1 protein level in tissue from heart and muscle. Data represent average ± SEM (n = 7 for each group). *$p < 0.05$ in an unpaired two-sample Student's t test.

(E) Average ± SEM body weight of 5-month-old male (n = 34 for each group) and female (n = 13 for each group) and of 9-month-old male (n = 18 for each group) and female (n = 16 for each group) C57/Bl6 littermates of the indicated genotype. *$p < 0.05$ in a two-tailed ANOVA test.

(F) Kaplan-Meier censorial analysis of C57/Bl6 mice of the indicated genotype (n = 30 male and n = 26 female littermates).

See also Figure S1.

2007), and uncontrolled OPA1 overexpression is toxic (Cipolat et al., 2004), complicating the generation of suitable in vivo models. To circumvent these difficulties, we recently generated a mouse model where a transgene carrying *Opa1* isoform 1 under the control of the ubiquitous human β-actin promoter was purposely targeted to a permissive X chromosome region, without altering endogenous gene expression (Cogliati et al., 2013). We capitalized on this mouse model to investigate the role of the OPA1-dependent cristae remodeling pathway in vivo. Our data indicate that mild OPA1 overexpression is compatible with life and blunts damage of highly metabolically active organs in response to apoptotic, necrotic, and atrophic stimuli by reducing cytochrome c release and mitochondrial dysfunction, thereby highlighting the importance of cristae shape in tissue homeostasis and non-developmental cell death.

RESULTS

Controlled OPA1 Overexpression Is Compatible with Embryonic Development and Does Not Affect Lifespan

Opa1-overexpressing mice (*Opa1^tg*) generated by targeting a single copy of murine *Opa1* isoform 1 driven by human β actin promoter immediately upstream of the mouse X chromosome *Hprt* locus (Cogliati et al., 2013) (Figures 1A and 1B) were backcrossed for more than 10 generations in C57/Bl6J and SV129 genetic backgrounds. *Opa1^tg* mice were born at the expected Mendelian frequency, developed and grew normally, and were apparently indistinguishable from sex- and age-matched wild-type (WT) littermates. The expected mild (~1.5-fold), but statistically significant, OPA1 overexpression was detected by western blot analysis in all the tissue tested (Figures 1C and 1D). Conversely, expression levels of the key mitochondrial fusion-fission proteins as well as of the pro- and anti-apoptotic Bcl-2 family proteins were unaltered (Figures S1A and S1B). While *Opa1^tg* and WT littermates' body weight was comparable during the first 60 days after birth (Figure S1C), in the obesity-prone C57/Bl6J strain (Almind and Kahn, 2004) the body weight of 5- to 9-month-old *Opa1^tg* individuals was slightly, but significantly, reduced in respect to their WT littermates (Figure 1E). This effect of OPA overexpression was not recorded in the Sv129 background (Figure S1D) and had no effect (beneficial or detrimental) on the overall survival as shown by a Kaplan-Meyer censorial analysis (Figures 1F and 1G). In conclusion, *Opa1^tg* mice are viable and display no apparent major phenotype, indicating that a mild OPA1 overexpression is compatible with life and fertility.

Physiological Heart Hypertrophy in 9-Month-Old *Opa1^tg* Mice

In order to address the long-term effects of OPA1 overexpression, we aged the *Opa1^tg* animals and analyzed morphologically and histologically several organs at different time points. Upon gross inspection, hearts of 9-month-old *Opa1^tg* mice appeared bigger than their WT littermates (Figure 2A). Accordingly, the heart/body weight ratio (Figure 2B) and the cardiomyocyte cross-sectional area (Figures 2C and 2D) were increased in 9-month-old *Opa1^tg* mice, but not in younger animals (Figures S2A and S2B). Since other parenchymal organs, such as liver and kidney, were not enlarged, as revealed by post-mortem as well as in vivo echography inspection (Figures S2D–S2F), we concluded that the hypertrophy was specific to the heart. The observed hypertrophy was not accompanied by echocardiographic signs of cardiac dysfunction at 9 (Figures 2E and 2F) as well as at 5

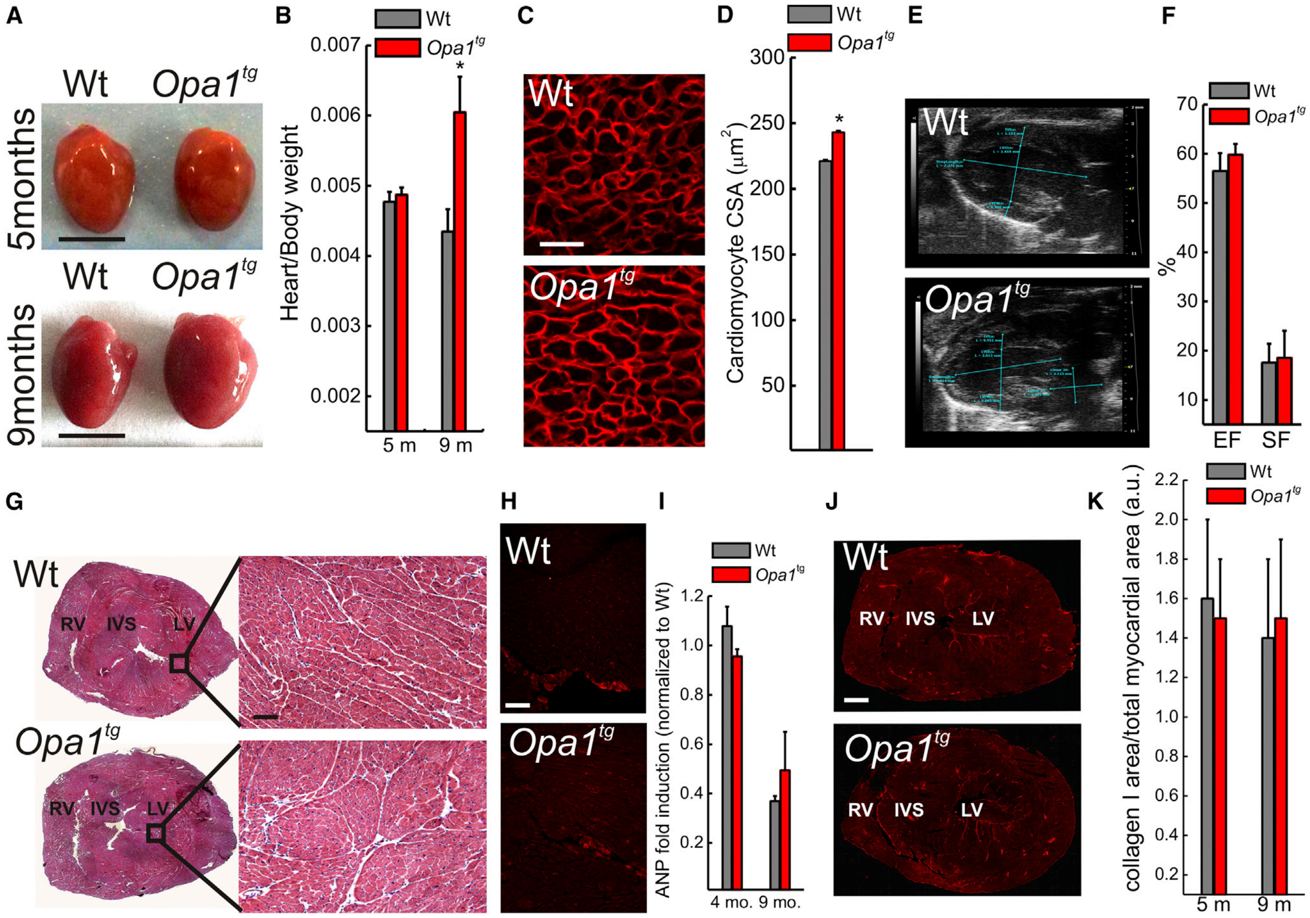

Figure 2. *Opa1*^{tg} Mice Develop a Non-pathological Cardiac Hypertrophy

(A) Representative photographs of hearts dissected from littermates of the indicated genotype and age. Scale bars, 0.5 cm.

(B) Average ± SEM heart/body weight ratio in littermates of the indicated genotype and age (n = 8 in each group). *p < 0.05 in a two-tailed ANOVA test versus 5 months *Opa1*^{tg} dataset.

(C) Representative detail confocal images of left ventricular cryosections stained for dystrophin from 9-month-old mice of the indicated genotype. Scale bar, 25 μm.

(D) Experiments were as in (C). Data represent mean ± SEM of four independent experiments. *p < 0.05 in an unpaired two-sample Student's t test.

(E) Echocardiographic long axis view of hearts from 9-month-old littermates of the indicated genotype. LV, left ventricle; A, aorta.

(F) Experiments were as in (E). Data represent mean ± SEM percentage of cardiac ejection fraction (EF) and shortening (SF) of four independent experiments.

(G) H&E staining of ventricular cryosections from 9-month-old littermates of the indicated genotype. Scale bars, 100 μm.

(H) Representative immunofluorescence images of ventricular cryosections stained for atrial natriuretic peptide (ANP) from 9-month-old littermates of the indicated genotype. Scale bar, 100 μm.

(I) Data represent average ± SEM of ANP mRNA levels determined by RT-PCR in 4- and 9-month-old littermates of the indicated genotype (n = 4 for each group).

(J) Representative immunofluorescence images of ventricular cryosections stained for collagen I from 9-month-old littermates of the indicated genotype. Scale bar, 100 μm.

(K) Experiments were as in (J). Data represent mean ± SEM of four independent experiments.

See also Figure S2.

(Figure S2G) months of age. Echocardiographic speckle tracking analysis to assess myocardial performance/load relationship excluded that *Opa1*^{tg} hearts contract against an increased afterload, indicative of increased blood pressure, a major cause of heart hypertrophy: the time to peak (TtPk) of myocardial shortening of longitudinal and radial strains were comparable in *Opa1*^{tg} and WT hearts (longitudinal strain TtPk, 81.6 ± 6.15 ms in *Opa1*^{tg} versus 63.0 ± 4.1 ms in WT; radial strain TtPk, 50.0 ± 33.4 ms in *Opa1*^{tg} versus 49.3 ± 20.1 ms in WT). All the tests for pathological hypertrophy were negative: histology of 9-month-old WT and *Opa1*^{tg} hearts was superimposable (Figure 2G), the fetal hypertrophy gene atrial natriuretic peptide (ANP) (Du, 2007) was not induced, as judged by immunofluorescence and RT-PCR analysis (Figures 2H and 2I), and hearts were also negative for the other hypertrophy gene β-myosin heavy chain (β-MHC) (Figure S2H). Furthermore, the lack of collagen-I immunostaining excluded interstitial fibrosis (Figures 2J and 2K) in 9-month-old *Opa1*^{tg} hearts. Finally, since mitochondrial dysmorphology and dysfunction characterize pathological cardiac hypertrophy (Abel and Doenst, 2011), we set out to investigate mitochondrial shape and activity in 9-month-old *Opa1*^{tg} hearts. Mitochondrial ultrastructure, evaluated by electron microscopy (EM; Figure S2I),

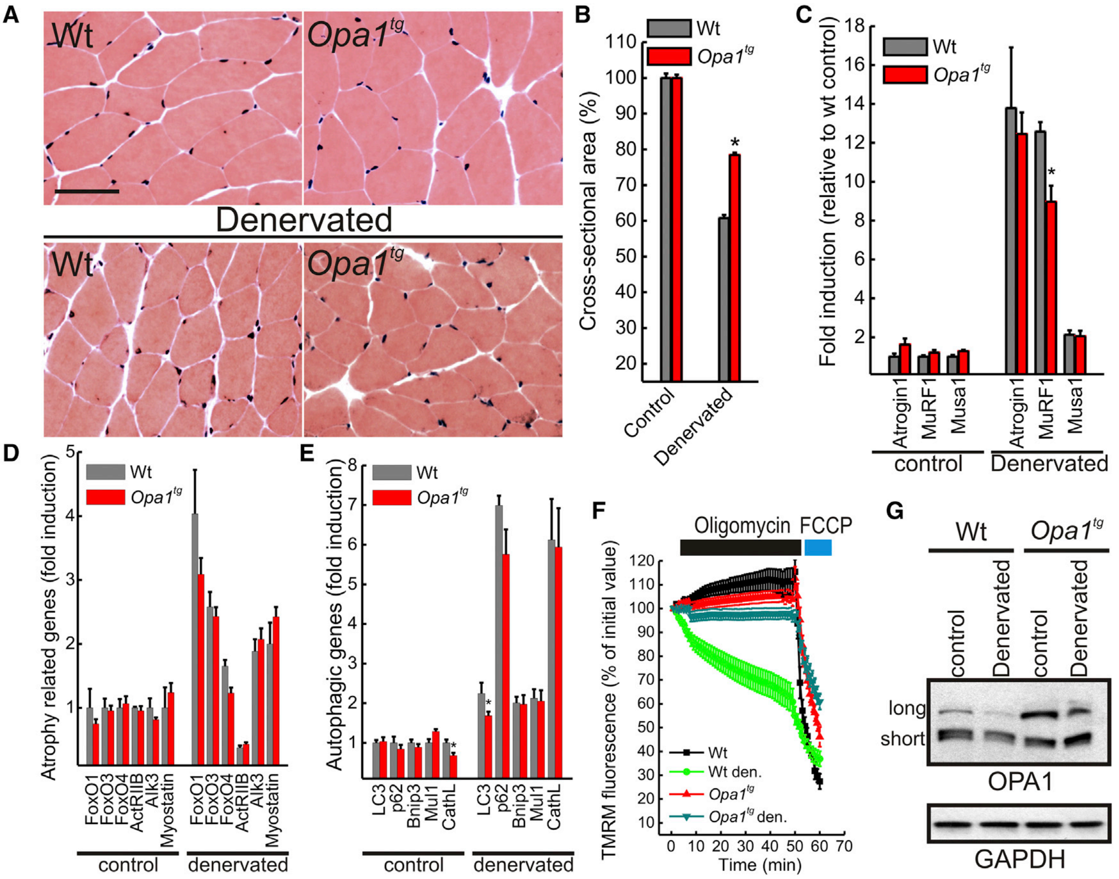

Figure 3. *Opa1^tg* Mice Are Protected from Denervation-Induced Muscle Atrophy

(A) H&E staining of control and 10-days-denervated gastrocnemial cryosections from 9-month-old littermates of the indicated genotype. Scale bars, 50 μm.

(B) Experiments were as in (A). Muscle loss 10 days after denervation was quantified by cross-sectional area (CSA) measurement of innervated and denervated fibers. Data represent mean ± SEM of five independent experiments. *$p < 0.05$ in an unpaired two-sample Student's t test.

(C–E) Data represent average ± SEM of the indicated mRNA levels determined by RT-PCR in littermates of the indicated genotype treated as indicated ($n = 5$ for each group). *$p < 0.05$ in an unpaired two-sample Student's t test.

(F) Data are average ± SEM of five independent experiments of TMRM fluorescence analysis over mitochondrial regions in fibers isolated from FDB of the indicated genotype treated as indicated.

(G) Equal amounts (30 μg) of protein from gastrocnemius isolated from littermates of the indicated genotype treated as indicated were separated by SDS-PAGE and immunoblotted using the indicated antibodies. Den., denervated.

See also Figure S3.

biogenesis, measured by real-time PCR analysis of mRNA levels of adenine translocase 1 (ANT1), controlled by the master biogenetic factor PGC1α (Figure S2J), as well as bioenergetics, indicated by biochemical and histological respiratory chain complexes (RCCs) activity measurements (Figures S2K and S2L), were not impaired. Complex III and IV activities were in fact increased, probably as a consequence of RCS stabilization by OPA1 (Cogliati et al., 2013). Thus, 9-month-old *Opa1^tg* animals display physiological heart hypertrophy, and indeed they perform slightly, but significantly, better than their WT littermates on a treadmill exercise test (Civiletto et al., 2015 [this issue of *Cell Metabolism*]).

Opa1^tg Mice Are Protected from Denervation-Induced Muscular Atrophy

Since cardiomyocyte size results from the balance between trophic and atrophic processes, we wished to investigate if OPA1 overexpression counteracted the atrophic program that in muscle activates autophagy and requires mitochondrial fragmentation (Romanello et al., 2010) and in heart is exacerbated during aging when it is accompanied by lipofuscinosis (De Meyer et al., 2010). We therefore turned to ischiectomy-induced muscle loss, an established model of muscle atrophy where mitochondria are targets of degradation and active participants in myonuclear apoptosis (Siu and Alway, 2005; Sandri et al., 2006; Adhihetty et al., 2007). Histological analysis did not reveal signs of inflammation, degeneration, or regeneration in gastrocnemius sections from control or 10-day-denervated 5-month-old WT and *Opa1^tg* mice (Figure 3A), but *Opa1^tg* mice were significantly protected from denervation-induced muscle mass loss (Figure 3B) in both oxidative or glycolytic fibers (Figure S3A). Accordingly, induction of Muscle Ring-Finger protein 1 (Murf1), the muscle-specific atrophy-related ubiquitin ligase that controls degradation of sarcomeric proteins (Sandri, 2013), was

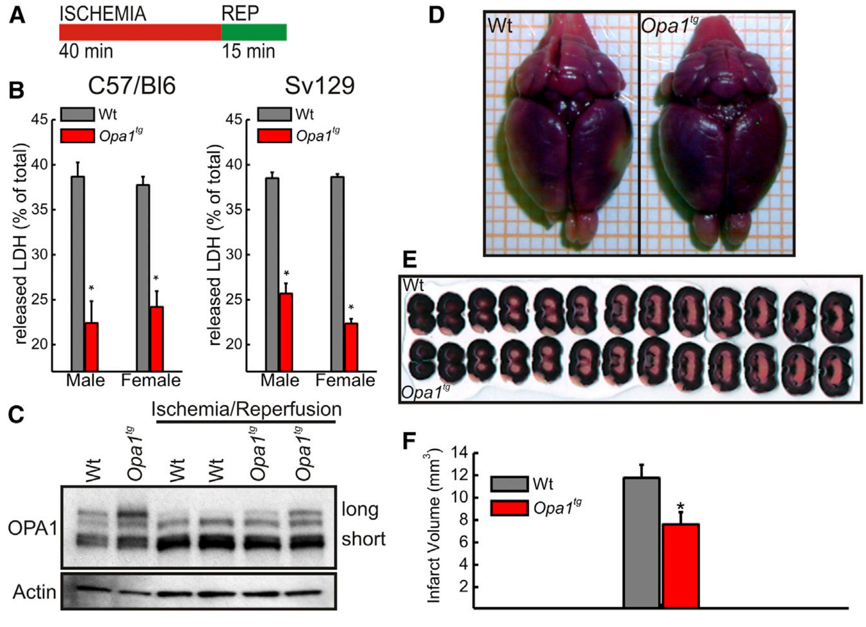

Figure 4. *Opa1*^{tg} Mice Are Protected from Ischemic Damage

(A) Schematic representation of the experimental ischemia-reperfusion protocol used.

(B) Data represent average ± SEM of eight independent experiments of lactate dehydrogenase (LDH) release during reperfusion in 5-month-old littermates of the indicated genotype, sex, and background (n = 8 for each group). $^{*}p < 0.05$ in a two-tailed ANOVA test.

(C) Equal amounts (30 μg) of heart protein from littermates of the indicated genotype treated as indicated were separated by SDS-PAGE and immunoblotted using the indicated antibodies.

(D) Representative images of TTC-stained whole brains from 3-month-old Sv129 female littermates of the indicated genotype mice 72 hr after middle cerebral artery occlusion (MCAo).

(E) Representative TTC-stained 250-μm brain sections from 3-month-old Sv129 female littermates of the indicated genotype 72 hr after MCAo. Slices are aligned from anterior to posterior.

(F) Data represent average ± SEM of four independent experiments carried out as in (E). $^{*}p < 0.05$ in an unpaired two-sample Student's t test.

reduced in denervated *Opa1*^{tg} muscles (Figure 3C), suggesting that OPA1 overexpression inhibits the atrophic program. Mechanistically, however, expression levels of atrophy-related genes of the ubiquitin-proteasome and autophagy-lysosome families that control myofiber size during disuse muscle atrophy (Powers et al., 2007; Sandri, 2008) were comparable in control and denervated WT and *Opa1*^{tg} muscles (Figures 3D and 3E), and autophagic flux was not altered in mouse adult fibroblasts (MAFs) derived from *Opa1*^{tg} mice (Figures S3B and S3C). We therefore investigated mitochondrial biogenesis and function in control and denervated muscles. Levels of the mitochondrial dynamics genes (except, of course, Opa1) and of the mitochondriobiogenetic factor Pgc1α were not changed (Figure S3D), suggesting that OPA1 overexpression does not induce biogenesis. Conversely, an established assay of mitochondrial dysfunction (Irwin et al., 2003) that characterizes muscle atrophy (Seo et al., 2010) revealed that *Opa1*^{tg} fibers were protected from denervation-induced mitochondrial dysfunction (Figure 3F). This assay reveals latent mitochondrial dysfunction and was more sensitive than histological and biochemical cytochrome c oxidase (COX) activity measurements, which resulted normal in gastrocnemius (Figures S3E and S3F) and in soleus (Figure S3G) in both genotypes, also after denervation. We could correlate the observed protection to the levels of the long OPA1 form that supports most of OPA1 functions (Anand et al., 2014) and that was almost completely degraded in denervated WT, but not *Opa1*^{tg}, muscles (Figure 3G; long OPA1 represented only ~4% of total OPA1 in WT, but ~17% in *Opa1*^{tg}-denervated muscles). Notably, the protection from muscular atrophy was long term, as shown by the preservation of muscular function and mass by OPA1 overexpression in a severe model of myopathy caused by skeletal muscle COX 15 (*Cox15*$^{sm/sm}$) ablation (see the accompanying manuscript by Civiletto et al. [2015] [this issue of *Cell Metabolism*]). Thus, OPA1 overexpression protects from acute and chronic muscle atrophy by blunting mitochondrial dysfunction and expression of the critical ubiquitin ligase MuRF1, without altering the autophagic program or inducing mitochondrial biogenesis.

Opa1^{tg} Mice Are Protected from Ischemic Damage

The protection of *Opa1*^{tg} mice from muscular atrophy prompted us to investigate other models of tissue damage where mitochondria play a central role. Myocardial infarction, besides being the leading cause of death in the Western world, is characterized by central necrosis and peripheral apoptosis, two processes in which mitochondrial dysfunction participates during both the ischemic and the reperfusion phases (Murphy and Steenbergen, 2008). By using a classical Langendorff-perfused heart preparation, we subjected hearts of 5-month-old mice to 40 min of ischemia followed by 15 min of reperfusion (I/R; Figure 4A) (Barbato et al., 1996) and assessed cardiac injury by measuring lactate dehydrogenase (LDH) release during the reperfusion period. OPA1 overexpression reduced the amount of released LDH in male and female C57/Bl6 and SV129 mice (Figure 4B), thereby excluding potentially confounding effects of genetic background and gender that influence the response to heart ischemia (Du, 2004). Similar to what was observed during skeletal muscle denervation, I/R was accompanied by long OPA1 degradation in WT, but not *Opa1*^{tg}, hearts (Figure 4C; long OPA1 represents only ~0.6% of total OPA1 in WT and ~10% in *Opa1*^{tg} hearts). The protection from ischemia was not confined to heart, since infarct volume, assessed in triphenyl tetrazolium chloride (TTC)-stained serial sections from brains 3 days after middle cerebral artery occlusion (MCAo) (Colak et al., 2011), was significantly reduced in *Opa1*^{tg} mice (Figures 4D–4F). These results indicate that mild OPA1 overexpression protects heart and brain from ischemic damage.

Opa1^{tg} Are Less Susceptible to Fas-Induced Liver Damage

The protection afforded by OPA1 from heart and brain ischemia corroborates early findings that implicated OPA1

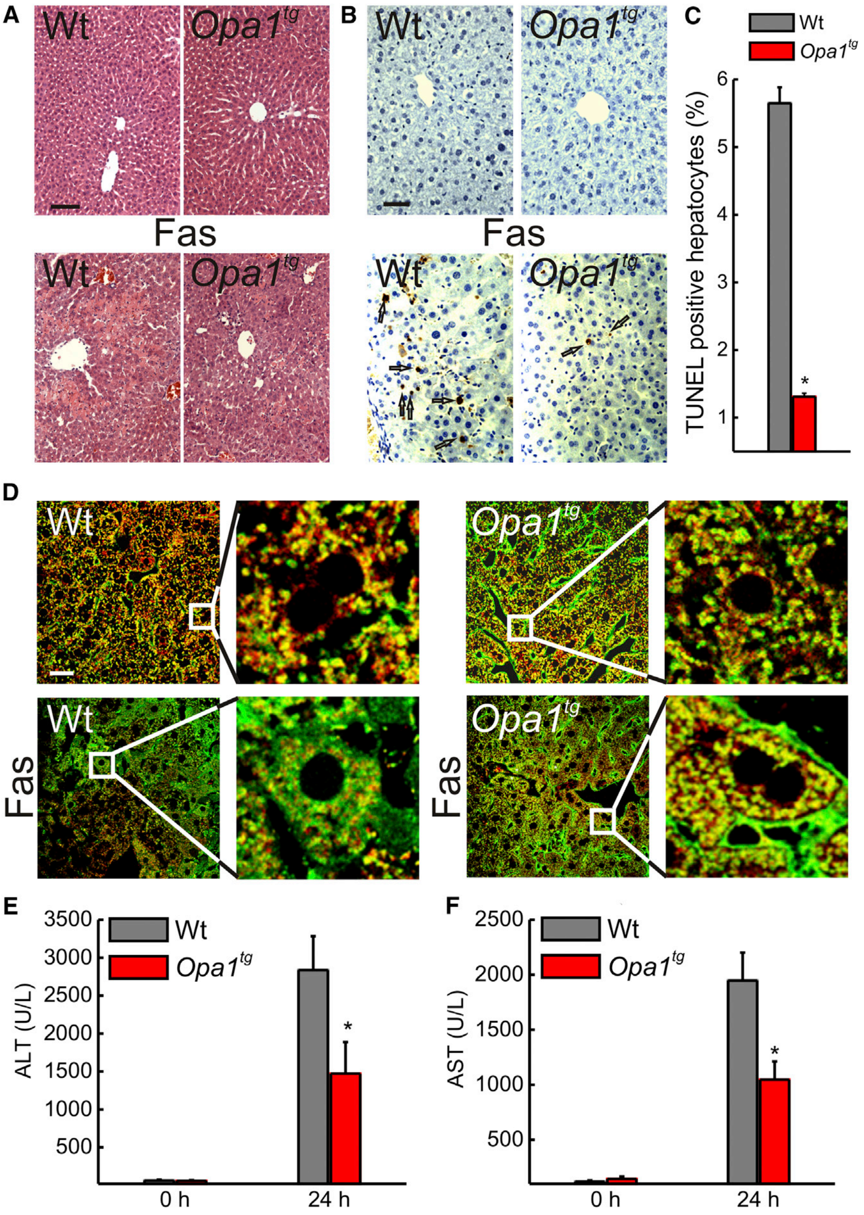

Figure 5. *Opa1^tg* Mice Are Protected from Fas-Induced Apoptotic Liver Damage

(A) Representative images of H&E-stained paraffin-embedded liver sections from littermates of the indicated genotype. Where indicated mice were tail vein injected with 0.25 μg/g anti-Fas antibody (Fas), sacrificed after 24 hr, and livers were explanted for histology. Scale bar, 50 μm.

(B) Experiments were as in (A), except that paraffin-embedded liver sections were TUNEL stained. Arrows indicate TUNEL-positive hepatocytes. Scale bar, 25 μm.

(C) Data represent average ± SEM of four independent experiments carried out as in (B). At least 30 images per group per experiment were analyzed. *p < 0.05 in an unpaired two-sample Student's t test.

(D) Representative confocal images of liver cryosections from littermates of the indicated genotypes treated as indicated and immunostained for cytochrome c (green) and TOM20 (red). The boxed areas are magnified 5×. Scale bar, 20 μm.

(E and F) Plasma ALT (E) or AST (F) levels in mice of the indicated genotypes at the indicated time points after tail vein injection of 0.25 μg/g anti-Fas antibody. Data represent average ± SEM (n = 7 in E, and n = 8 in F, for each group). *p < 0.05 in an unpaired ANOVA test.

See also Figure S4.

and OPA1-dependent cristae remodeling in cell death. However, we wished to further challenge this hypothesis by turning to a model of in vivo apoptosis. Fas receptor activation in the liver by means of tail vein injection of anti-Fas-activating antibodies is a well-characterized model of severe apoptotic liver damage (Ogasawara et al., 1993) where Fas-induced death is dependent on mitochondria (Yin et al., 1999; Wei et al., 2000). While liver histology was not different in 3-month-old BL6 WT and *Opa1^tg* mice, 24 hr after tail vein injection of an activating anti-Fas antibody, damage was greatly decreased in *Opa1^tg* livers, where the lobular architecture was preserved and the hemorrhagic infiltrates reduced (Figure 5A). Accordingly, TUNEL staining revealed a significant reduction in apoptotic cell death in *Opa1^tg* livers (Figures 5B and 5C). Also, Sv129 *Opa1^tg* mice were less susceptible than their WT littermates to the same anti-Fas antibody-induced liver damage, as judged by survival and liver histology (Figure S4). Mechanistically, the protection afforded by OPA1 correlated with the inhibition of cytochrome c release from mitochondria, as revealed by the analysis of subcellular cytochrome c distribution in Fas-treated WT and *Opa1^tg* livers (Figure 5D). Consistently, the increase in plasma alanine and aspartate transaminases (ALT and AST) levels, a liver damage indicator, was significantly lower in Fas-treated *Opa1^tg* mice versus their WT-treated littermates (Figures 5E and 5F). Thus, OPA1 overexpression inhibits mitochondrial-dependent hepatocyte apoptosis in vivo.

Opa1^tg Mitochondria Are Resistant to Cristae Remodeling and Cytochrome C Release

We next wished to address mechanistically how OPA1 overexpression protected from these multiple and different death stimuli in vivo. In immortalized fibroblasts, OPA1 modulates mitochondrial apoptosis (Frezza et al., 2006) as well as RCS assembly and mitochondrial respiration efficiency (Cogliati et al., 2013). We therefore sought to verify if the same held true in primary tissues. As expected, mitochondria were longer in primary myoblasts prepared from *Opa1^tg* diaphragms (Figures 6A and 6B), and cristae tighter in hearts (Figures 6C and 6D) as well as in other organs (data not shown) from *Opa1^tg* mice. Functionally, *Opa1^tg* primary fibroblasts produced less mitochondrial reactive

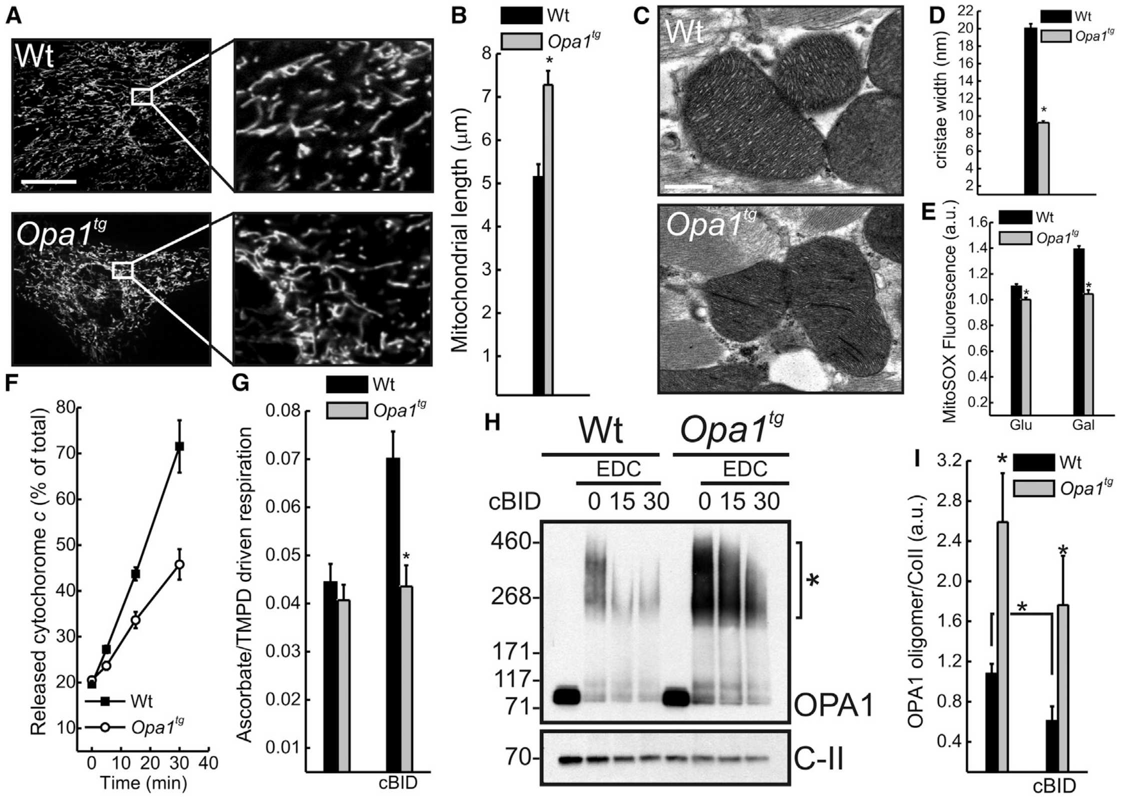

Figure 6. *Opa1*^{tg} Mitochondria Are Resistant to Apoptotic Cristae Remodeling and Cytochrome C Release

(A) Representative confocal images of mitochondrially targeted YFP transfected in primary myoblasts of the indicated genotype. Scale bar, 20 μm.

(B) Data represent average ± SEM of four independent experiments (30 cells/experiment) of mitochondrial major axis length calculation in experiments as in (A). *$p < 0.05$ in an unpaired two-sample Student's t test.

(C) Representative electron micrographs of hearts of the indicated genotype. Scale bar, 500 nm.

(D) Morphometric analysis of cristae width in 80 randomly selected mitochondria of 5-month-old hearts from WT and *Opa1*^{tg} mice. *$p < 0.05$ in an unpaired two-sample Student's t test.

(E) Fold increase in MitoSOX fluorescence after treatment with antimycin A (10 μM, 20 min) in cells incubated for 24 hr in DMEM supplemented with the indicated monosaccharides. Data represent average ± SEM of four independent experiments. *$p < 0.05$ in an unpaired two-sample Student's t test.

(F) Mitochondria isolated from livers of the indicated genotypes were treated for the indicated times with 40 pmol/mg cBID, and cytochrome c release was measured by ELISA. Data represent average ± SEM of five independent experiments.

(G) Ascorbate/TMPD-driven respiration of purified liver mitochondria of the indicated genotypes treated where indicated for 15 min with cBID. Data represent average ± SEM of four independent experiments. *$p < 0.05$ in an unpaired two-sample Student's t test.

(H) Purified muscle mitochondria of the indicated genotype were treated for the indicated times with cBID, and, where indicated, 10 mM EDC was added. Equal amounts (30 μg) of proteins were separated by SDS-PAGE and immunoblotted using the indicated antibodies.

(I) Densitometric analysis of OPA1 oligomers. Experiments were as in (H). Data represent average ± SEM of five independent experiments. *$p < 0.05$ in a two-way ANOVA with the corresponding time point WT or between the indicated samples.

See also Figure S5.

oxygen species (ROS) when supplied with excess glucose or galactose that forces mitochondrial respiration (Figure 6E). This reduction of ROS production was not caused by desensitization of the mitochondrial permeability transition pore (PTP) that aggravates ROS production and apoptosis (Basso et al., 2005), since complex IV-dependent mitochondrial respiration (Figure S5A), membrane potential (Figure S5B), and basal and maximal calcium retention capacity (CRC; a sensitive indicator of PTP threshold; Figures S5C and S5D) were superimposable in WT and *Opa1*^{tg} mitochondria. Conversely, respiratory control ratio (RCR) of *Opa1*^{tg} liver and fibroblast mitochondria energized with glutamate/malate is higher (Cogliati et al., 2013), supporting reduced ROS production from stabilized RCSs. In addition to reduced ROS production, *Opa1*^{tg} mitochondria were releasing less cytochrome c in an established assay of mitochondrial permeabilization based on recombinant active caspase-cleaved BID (cBID) (Scorrano et al., 2002; Frezza et al., 2006) (Figure 6F), notwithstanding that BAK activation (measured as its oligomerization) in response to cBID was comparable in WT and *Opa1*^{tg} mitochondria (Figure S5E). To verify if cytochrome c mobilization from the cristae was blunted in *Opa1*^{tg} mitochondria we measured the ratio of ascorbate (asc)-driven over tetramethyl-p-phenylenediamine (TMPD)-driven respiration, an established assay of submitochondrial cytochrome c localization (Scorrano et al., 2002). While basal asc/TMPD ratios were comparable, cBID increased it only in WT mitochondria (Figure 6G), indicating

that the apoptotic redistribution of cytochrome c was blocked in *Opa1*^{tg} mitochondria. Accordingly, the OPA1 oligomers targeted by cBID to trigger cristae remodeling and cytochrome c redistribution (Frezza et al., 2006) were stabilized in *Opa1*^{tg} mitochondria (Figures 6H, 6I, and S5E). Taken together these results indicate that mild OPA1 overexpression blunts apoptotic cristae remodeling in vivo.

DISCUSSION

The field of mitochondrial dynamics blossomed when the processes of cristae remodeling and mitochondrial fragmentation during apoptosis were discovered (Scorrano et al., 2002; Frank et al., 2001; Martinou et al., 1999). The discovery that OPA1 controls cristae remodeling (Frezza et al., 2006) and RCS assembly and stability (Cogliati et al., 2013) offered a conceptual framework to explore whether OPA1 overexpression interfered with cell death in vivo. Our results indicate that mild OPA1 overexpression is compatible with life and protects mice from multiple form of damage, implicating cristae remodeling in myocardial and brain infarction, hepatocellular apoptosis, and muscular atrophy.

In order to investigate the role of cristae remodeling in vivo we elected to modulate levels of its master regulator OPA1. Not surprisingly, given the crucial role of this protein in mitochondrial physiology, its ablation is lethal early during intrauterine development (Davies et al., 2007), and its inducible deletion in differentiated tissues such as brain and skeletal muscle is similarly fatal (data not shown). We therefore turned to a different approach and generated a mouse model of targeted, controlled *Opa1* overexpression (Cogliati et al., 2013) where we could avoid the paradoxical toxic effects of high OPA1 levels (Cipolat et al., 2004).

Opa1 overexpression does not impair fertility, intrauterine development, adult life, and lifespan, a surprising lack of effect for a gene with a documented anti-apoptotic effect. Conversely, in the BL6 background it slightly, but significantly, lowered body weight at 5–9 months, and in both BL6 and Sv129 backgrounds it granted protection from a panoply of insults to different tissues. It shall be mentioned, however, that in the Sv129 background the incidence of spontaneous cancer is increased, and lifespan is accordingly reduced (data not shown), unlike the crucial apoptosis inhibitors *Bcl*-2 and *Bcl*-X_L whose overexpression does not promote cancer per se, except when it is confined in the B cell compartment (Frenzel et al., 2009; Pena et al., 1998). The small effect of *Opa1* overexpression on body weight might be a consequence of its role on mitochondrial bioenergetics, or it could result from a yet-unclear effect of cristae shape on intermediate metabolism. In any case, it suggests that the relationship between cristae shape and weight control shall be addressed experimentally. Finally, the increase in cancer prevalence offers a potential explanation as to why animals do not display constitutively high OPA1 levels that are conversely beneficial against adult life cell death.

Chronic muscle disuse induced by denervation results in muscle atrophy with accompanying mitochondrial alterations (Adhihetty et al., 2007) and network disruption (Romanello et al., 2010). The anti-atrophic effect of OPA1 overexpression was not confined to acute muscle loss caused by denervation, but also counteracted chronic muscle loss and myopathy caused by a genetic mitochondrial defect (Civiletto et al., 2015 [this issue of *Cell Metabolism*]), further substantiating the key role of OPA1 in the control of muscle atrophy. Mechanistically, OPA1 overexpression did not stimulate mitochondrial biogenesis or blunted autophagy, but it sustained mitochondrial function. OPA1 expression indeed triggered a slight mitochondrial elongation, which was accompanied by a remarkable increase in respiratory capacity that correlated with cristae tightness (Cogliati et al., 2013). Thus, maintenance of mitochondrial cristae seems a feasible strategy to counteract muscular atrophy, an emerging medical problem in the aging society.

In addition to skeletal muscle, brain and heart are also high-energy-demanding tissues where mitochondrial dysfunction is detrimental. Indeed, ischemia and I/R injury are associated with a dramatic change in mitochondrial morphology and ultrastructure (Calo et al., 2013). In addition to its role in apoptosis, mitochondrial dynamics has recently been implied in necrosis and necroptosis (Orogo and Gustafsson, 2013; Wang et al., 2012). Mild OPA1 overexpression was sufficient to improve enzymatic parameters associated with tissue damage in response to ischemic injury. Our results suggest that cristae remodeling is central also to propagate necrotic damage, and that its prevention can ameliorate the ischemic damage outcome. Notably, OPA1 overexpression did not increase brain or heart vascularization (data not shown), suggesting a direct effect on the mechanisms of ischemic cell damage. During muscle atrophy as well as heart I/R, long OPA1 forms appear to be cleaved, presumably by the stress-activated ATP-independent metalloprotease OMA1 (Baker et al., 2014), to inactivate OPA1 function that depends in full (Anand et al., 2014) or at least partially (Cipolat et al., 2006) on its long membrane inserted forms. Since stabilization of long OPA1 in *Opa1*^{tg} mice was associated with I/R and muscular atrophy protection, it is tempting to speculate that OPA1 cleavage inhibitors could be cardio and muscle protective. Finally, the importance of OPA1 in brain and muscle is further corroborated by the finding that its overexpression ameliorates two mouse models of mitochondrial diseases characterized by profound neurological and muscular defects (Civiletto et al., 2015 [this issue of *Cell Metabolism*]).

Opa1^{tg} hearts at 9 months are hypertrophic. Our analysis failed to reveal any histological or functional sign of maladaptive hypertrophy or of increased afterload, one of the most common causes of hypertrophy. We can speculate that *Opa1* overexpression results in physiological hypertrophy because it blunts normal atrophic processes also in the heart, or that the improved *Opa1*^{tg} mouse motor performance results in an endurance training type of hypertrophy, or that the ameliorated *Opa1*^{tg} mitochondrial function directly results in increased myocardial mass, directly linking mitochondrial function to physiological hypertrophy. Irrespective of its underlying mechanism, the observed hypertrophy does not account for *Opa1*^{tg} heart protection from I/R, measured in isolated preparations and before its onset.

The liver, where Fas is constitutively expressed on hepatocytes (Ogasawara et al., 1993), is very sensitive to Fas-mediated apoptosis. Because hepatocytes are type II cells in which Fas-induced death is dependent on the mitochondrial pathway, Fas receptor activation in the liver has been widely employed to study apoptosis in vivo (Rodriguez et al., 1996; Yin et al.,

1999; Wei et al., 2001). Notably, OPA1 overexpression also protected from Fas-mediated hepatocellular apoptosis. While OPA1 expression did not interfere with Bax, Bak mediated outer membrane permeabilization, and it blunted cytochrome c release in vitro and in vivo by counteracting its intramitochondrial redistribution during cristae remodeling.

Our work unravels a role for OPA1 and mitochondrial cristae remodeling in multiple forms of cell death in vivo. The analysis of a mouse model of mild, targeted OPA1 overexpression extends the types of cell death controlled by cristae remodeling beyond apoptosis, indicates a crucial role for mitochondrial ultrastructure in tissue adaptation to pathological stimuli, and offers a proof of principle for therapies aimed at ameliorating mitochondrial cristae in acute and chronic diseases.

EXPERIMENTAL PROCEDURES

Generation of *Opa1^{tg}* Mice

Experimental procedures for the generation of *Opa1^{tg}* mice were described (Cogliati et al., 2013). *Opa1^{tg}* mice were initially developed in a mixed genetic background of C57BL/6 × Sv129Ola and were backcrossed to C57BL/6 and Sv129 for more than ten generations. The genotype was determined by analysis of DNA extracted from tail biopsies via PCR. All procedures were authorized by the Office Veterinaire Cantonal of Geneva (1034/3703/2 to L.S.) and the CEASA of the University of Padova (32/2011 to L.S.).

Echocardiography

Echocardiography was performed in adult (5 and 9 months) *Opa1^{tg}* (n = 6) and age- and sex-matched littermate controls (n = 6) using a Vevo 2100 (VisualSonics, Toronto) system equipped with a 30-MHz transducer. Anesthesia was induced with 3% isoflurane, maintained with 1.5% isoflurane during constant monitoring of temperature, respiration rate, and ECG. Details on echocardiography analysis can be found in Supplemental Experimental Procedures.

Muscle Denervation

Denervation was performed in 5-month-old males by cutting the left hind limb sciatic nerve, with the right hind limb being used as control. At 10 days after denervation animals were sacrificed by cervical dislocation, and muscles were utilized for histological experiments or gene expression studies.

Heart I/R

Heart I/R was performed using a nonrecirculating Langendorff model (Barbato et al., 1996). The hearts were rapidly excised from cervical-dislocated 5 months sex-matched animals and perfused retrogradely for 10 min with a bicarbonate buffer (118.5 mM NaCl, 3.1 mM KCl, 1.18 mM KH_2PO_4, 25.0 mM $NaHCO_3$, 1 mM $MgCl_2$, 1.4 mM $CaCl_2$, and 5.6 mM glucose) insufflated with 37°C 95% O_2-5% CO_2 (pH 7.4) at a constant flux of 5 ml/min. After 40 min of ischemia, hearts were reperfused for 15 min. Coronary effluent during the reperfusion period was collected to measure LDH. At the end of reperfusion the hearts were quickly immersed into PBS supplemented with 0.5% Triton X-100 and homogenized for total LDH measurement.

Brain Ischemia

Animals were sacrificed 3 days after middle cerebral artery (MCA) occlusion. MCA ligation surgery in mice and 2,3,5-Triphenyltetrazolium chloride (TTC) staining for quantification of infarct volumes were performed as described (Colak et al., 2011).

Fas-Induced Apoptosis

Male littermates 3 months old of the indicated genotypes were tail vein injected with an activating anti-Fas antibody (JO2, 0.25 μg/g body weight in sterile saline solution, BD Pharmingen) or with sterile saline solution as a control. The animals were monitored and sacrificed 24 hr after injection for liver biochemistry or histology and clinical chemistry. Blood samples were collected in BD Microtainer PST LH Tubes and centrifuged at 6,000 g for 10 min to separate plasma. Plasma levels of alanine aminotransferase (ALT) and aspartate aminotransferase (AST) were determined using standard, Clinical Pathology Accredited and ISO9001:2008-certified procedures at the Department of Laboratory Medicine, Padova University Hospital. Liver samples were immediately frozen in liquid nitrogen for biochemical analysis or processed to paraformaldehyde (PFA) fixation for subsequent histological analysis and TUNEL staining.

Histology and Immunofluorescence Staining

Heart, muscle, and liver tissues were fixed in 1% PFA in PBS at room temperature for 15 min, equilibrated in a sucrose gradient, frozen in liquid nitrogen, sectioned, and processed for histological and immunofluorescence analyses. Sections (10 microns) were obtained with a cryostat (Leica CM1850, Leica Microsystems, Wetzlar) and incubated with primary antibodies diluted in PBS supplemented with 1% BSA and 0.5% Triton X-100 overnight at 4°C. Details on the antibodies used can be found in Supplemental Experimental Procedures. Livers were also cut into small pieces and fixed overnight at 4°C in 4% PFA in PBS and then dehydrated through serial ethanol concentrations for paraffin embedding. Paraffin-embedded livers were used for TUNEL and H&E staining. TUNEL assay was performed by using the ApopTag peroxidase in situ apoptosis detection system, following the procedure suggested by the manufacturer (Chemicon). For COX and SDH histochemical analysis, tissues were frozen in liquid nitrogen pre-cooled isopentane, and 8-μm-thick sections were stained as described (Sciacco and Bonilla, 1996).

Biochemical Analysis of MRC Complexes

Skeletal and cardiac muscle samples were snap-frozen in liquid nitrogen and homogenized in 10 mM phosphate buffer (pH 7.4). The spectrophotometric activities of CI, CII, CIII, and CIV, as well as citrate synthase (CS), were measured as described (Bugiani et al., 2004).

Gene Expression Analyses

Total RNA was prepared from gastrocnemius muscles using TRIzol (Invitrogen). cDNA was generated from 0.4 μg of RNA reverse-transcribed with SuperScript III Reverse Transcriptase (Invitrogen). Duplicates of cDNA samples were then amplified on the 7900HT Fast Real-Time PCR System (Applied Biosystems) using the Power SYBR Green RT-PCR kit (Applied Biosystems). All data were normalized to GAPDH expression and plotted in arbitrary units as mean ± SEM of independent experiments. The oligonucleotide primers used can be found in Supplemental Experimental Procedures.

Assays of Mitochondrial Membrane Potential

Mitochondrial membrane potential in isolated flexor digitorum brevis (FDB) muscle fibers was measured by intensity of TMRM fluorescence. Details can be found in Supplemental Experimental Procedures.

In Vitro Mitochondrial Assays

Liver and muscle mitochondria were isolated as described (Frezza et al., 2007). Cytochrome c redistribution and release in response to 40 pmol cBID/mg mitochondria was performed as described (Scorrano et al., 2002). Mitochondrial oxygen consumption was measured with a Clark type oxygen electrode (Hansatech Instruments) as described (Frezza et al., 2007). Details on mitochondria Ca^{2+} retention capacity and mitochondrial membrane potential measurements can be found in Supplemental Experimental Procedures.

Biochemistry

For protein crosslinking, mitochondria were treated as indicated with 1 mM EDC or 1 mM BMH as described (Frezza et al., 2006). For SDS-PAGE, equal amounts of mitochondrial proteins were separated on 3%–8% Tris-acetate or 4%–12% Tris-MES (NuPAGE, Invitrogen) polyacrilamide gel, transferred onto PVDF membranes (Bio-Rad), and probed using the indicated primary antibodies and isotype-matched secondary antibodies conjugated to horseradish peroxidase. Signal was detected with ECL (Amersham). Details on the antibodies used can be found in Supplemental Experimental Procedures. Densitometry was performed using ImageJ.

To detect OPA1 oligomers by BN-PAGE, mitochondria were resuspended in an appropriated volume of Buffer D (1 M 6-aminohexanoic acid, 1.25% V/V digitonin, 50 mM Bis-Tris-HCl [pH 7]) at a final concentration of 10 mg/ml.

Following centrifugation, the supernatant was collected, and 5% Serva Blue G dye in 1 M 6-aminohexanoic acid was added to one-third of the final volume of the sample. Equal amounts (100 μg) of mitochondrial proteins were separated on a 3%–12% gradient BNGE (Invitrogen) as described (Schägger, 1995).

Determination of ROS Production

Mitochondrial ROS levels were analyzed in cells incubated for 24 hr in DMEM containing glucose (4.5 mg/ml) or galactose (0.9 mg/ml). Cells were stained with 2 μM MitoSOX (Invitrogen) in HBSS for 30 min at 37°C and rinsed, and the fluorescence was assessed by flow cytometry using the FL1 channel of a FACSCalibur (BD Pharmingen) cytometer.

Transmission EM

Heart specimens from mice of the indicated genotype were fixed in 2% formaldehyde, 2.5% (V/V) glutaraldehyde in 0.1 M Na-cacodylate (pH 7.4) for 2 hr at room temperature and then overnight at 4°C. EM was performed as described (Scorrano et al., 2002). Thin sections were imaged on a Tecnai-20 electron microscope (Philips-FEI).

SUPPLEMENTAL INFORMATION

Supplemental Information includes five figures and Supplemental Experimental Procedures and can be found with this article online at http://dx.doi.org/10.1016/j.cmet.2015.05.007.

AUTHOR CONTRIBUTIONS

T.V. performed experiments, analyzed data, and wrote the manuscript; M.E.S. generated the Opa1^{tg} colony and performed experiments; V.R. analyzed denervated muscles; T.Z. analyzed hearts; R.Q.-C. performed ROS measurements; M. Semenzato performed RT-PCR analyses; R.M. helped perform heart ischemia reperfusion; V.C. performed mitochondrial in vitro assays; G.C. performed respiratory chain activity staining; P.P. helped perform echocardiography; C.V., M.Z., F.D.L., M.M., and M. Sandri supervised research, analyzed data, and contributed to writing; and L.S. conceived the project, supervised research, analyzed data, and wrote the manuscript.

ACKNOWLEDGMENTS

We thank Drs. M. Zaninotto and R. Venturini (Deptartment of Laboratory Medicine, University of Padova) for ALT and AST measurements, Drs. F. Caicci and F. Boldrin (Department of Biology, University of Padova) for EM sample preparation, Dr. S. Cogliati (CSIC, Madrid) for helpful discussions, and Dr. E. Schrepfer (Department of Biology, University of Padova) for the graphical abstract preparation. L.S. is a Senior Scientist of the Dulbecco-Telethon Institute. This work was supported by Telethon-Italy GPP10005, GGP12162, GGP14187A; AIRC Italy, ERC FP7-282280, FP7 CIG PCIG13-GA-2013-618697; Italian Ministry of Research, FIRB RBAP11Z3YA_005; Swiss National Foundation, 31-118171 to L.S.; and ERC FP7-282310, Fondation Leducq to M. Sandri.

Received: September 30, 2014
Revised: February 18, 2015
Accepted: May 4, 2015
Published: June 2, 2015

REFERENCES

Abel, E.D., and Doenst, T. (2011). Mitochondrial adaptations to physiological vs. pathological cardiac hypertrophy. Cardiovasc. Res. *90*, 234–242.

Adhihetty, P.J., O'Leary, M.F.N., Chabi, B., Wicks, K.L., and Hood, D.A. (2007). Effect of denervation on mitochondrially mediated apoptosis in skeletal muscle. J. Appl. Physiol. *102*, 1143–1151.

Almind, K., and Kahn, C.R. (2004). Genetic determinants of energy expenditure and insulin resistance in diet-induced obesity in mice. Diabetes *53*, 3274–3285.

Anand, R., Wai, T., Baker, M.J., Kladt, N., Schauss, A.C., Rugarli, E., and Langer, T. (2014). The i-AAA protease YME1L and OMA1 cleave OPA1 to balance mitochondrial fusion and fission. J. Cell Biol. *204*, 919–929.

Baker, M.J., Lampe, P.A., Stojanovski, D., Korwitz, A., Anand, R., Tatsuta, T., and Langer, T. (2014). Stress-induced OMA1 activation and autocatalytic turnover regulate OPA1-dependent mitochondrial dynamics. EMBO J. *33*, 578–593.

Barbato, R., Menabò, R., Dainese, P., Carafoli, E., Schiaffino, S., and Di Lisa, F. (1996). Binding of cytosolic proteins to myofibrils in ischemic rat hearts. Circ. Res. *78*, 821–828.

Basso, E., Fante, L., Fowlkes, J., Petronilli, V., Forte, M.A., and Bernardi, P. (2005). Properties of the permeability transition pore in mitochondria devoid of Cyclophilin D. J. Biol. Chem. *280*, 18558–18561.

Bugiani, M., Invernizzi, F., Alberio, S., Briem, E., Lamantea, E., Carrara, F., Moroni, I., Farina, L., Spada, M., Donati, M.A., et al. (2004). Clinical and molecular findings in children with complex I deficiency. Biochim. Biophys. Acta *1659*, 136–147.

Calo, L., Dong, Y., Kumar, R., Przyklenk, K., and Sanderson, T.H. (2013). Mitochondrial dynamics: an emerging paradigm in ischemia-reperfusion injury. Curr. Pharm. Des. *19*, 6848–6857.

Cereghetti, G.M., Stangherlin, A., Martins de Brito, O., Chang, C.R., Blackstone, C., Bernardi, P., and Scorrano, L. (2008). Dephosphorylation by calcineurin regulates translocation of Drp1 to mitochondria. Proc. Natl. Acad. Sci. USA *105*, 15803–15808.

Chen, H., Detmer, S.A., Ewald, A.J., Griffin, E.E., Fraser, S.E., and Chan, D.C. (2003). Mitofusins Mfn1 and Mfn2 coordinately regulate mitochondrial fusion and are essential for embryonic development. J. Cell Biol. *160*, 189–200.

Cipolat, S., Martins de Brito, O., Dal Zilio, B., and Scorrano, L. (2004). OPA1 requires mitofusin 1 to promote mitochondrial fusion. Proc. Natl. Acad. Sci. USA *101*, 15927–15932.

Cipolat, S., Rudka, T., Hartmann, D., Costa, V., Serneels, L., Craessaerts, K., Metzger, K., Frezza, C., Annaert, W., D'Adamio, L., et al. (2006). Mitochondrial rhomboid PARL regulates cytochrome c release during apoptosis via OPA1-dependent cristae remodeling. Cell *126*, 163–175.

Civiletto, G., Varanita, T., Cerutti, R., Gorletta, T., Barbaro, S., Marchet, S., Lamperti, C., Viscomi, C., Scorrano, L., and Zeviani, M. (2015). Opa1 overexpression ameliorates the phenotype of two mitochondrial disease mouse models. Cell Metab. *21*, this issue, 845–854.

Cogliati, S., Frezza, C., Soriano, M.E., Varanita, T., Quintana-Cabrera, R., Corrado, M., Cipolat, S., Costa, V., Casarin, A., Gomes, L.C., et al. (2013). Mitochondrial cristae shape determines respiratory chain supercomplexes assembly and respiratory efficiency. Cell *155*, 160–171.

Colak, G., Filiano, A.J., and Johnson, G.V. (2011). The application of permanent middle cerebral artery ligation in the mouse. J. Vis. Exp. http://dx.doi.org/10.3791/3039.

Danial, N.N., and Korsmeyer, S.J. (2004). Cell death: critical control points. Cell *116*, 205–219.

Danial, N.N., Gramm, C.F., Scorrano, L., Zhang, C.Y., Krauss, S., Ranger, A.M., Datta, S.R., Greenberg, M.E., Licklider, L.J., Lowell, B.B., et al. (2003). BAD and glucokinase reside in a mitochondrial complex that integrates glycolysis and apoptosis. Nature *424*, 952–956.

Davies, V.J., Hollins, A.J., Piechota, M.J., Yip, W., Davies, J.R., White, K.E., Nicols, P.P., Boulton, M.E., and Votruba, M. (2007). Opa1 deficiency in a mouse model of autosomal dominant optic atrophy impairs mitochondrial morphology, optic nerve structure and visual function. Hum. Mol. Genet. *16*, 1307–1318.

De Meyer, G.R.Y., De Keulenaer, G.W., and Martinet, W. (2010). Role of autophagy in heart failure associated with aging. Heart Fail. Rev. *15*, 423–430.

DiMauro, S., and Schon, E.A. (2003). Mitochondrial respiratory-chain diseases. N. Engl. J. Med. *348*, 2656–2668.

Du, X.J. (2004). Gender modulates cardiac phenotype development in genetically modified mice. Cardiovasc. Res. *63*, 510–519.

Du, X.J. (2007). Divergence of hypertrophic growth and fetal gene profile: the influence of beta-blockers. Br. J. Pharmacol. *152*, 169–171.

Frank, S., Gaume, B., Bergmann-Leitner, E.S., Leitner, W.W., Robert, E.G., Catez, F., Smith, C.L., and Youle, R.J. (2001). The role of dynamin-related protein 1, a mediator of mitochondrial fission, in apoptosis. Dev. Cell *1*, 515–525.

Frenzel, A., Grespi, F., Chmelewskij, W., and Villunger, A. (2009). Bcl2 family proteins in carcinogenesis and the treatment of cancer. Apoptosis *14*, 584–596.

Frey, T.G., and Mannella, C.A. (2000). The internal structure of mitochondria. Trends Biochem. Sci. *25*, 319–324.

Frezza, C., Cipolat, S., Martins de Brito, O., Micaroni, M., Beznoussenko, G.V., Rudka, T., Bartoli, D., Polishuck, R.S., Danial, N.N., De Strooper, B., and Scorrano, L. (2006). OPA1 controls apoptotic cristae remodeling independently from mitochondrial fusion. Cell *126*, 177–189.

Frezza, C., Cipolat, S., and Scorrano, L. (2007). Organelle isolation: functional mitochondria from mouse liver, muscle and cultured fibroblasts. Nat. Protoc. *2*, 287–295.

Griparic, L., and van der Bliek, A.M. (2001). The many shapes of mitochondrial membranes. Traffic *2*, 235–244.

Hackenbrock, C.R. (1966). Ultrastructural bases for metabolically linked mechanical activity in mitochondria. I. Reversible ultrastructural changes with change in metabolic steady state in isolated liver mitochondria. J. Cell Biol. *30*, 269–297.

Irwin, W.A., Bergamin, N., Sabatelli, P., Reggiani, C., Megighian, A., Merlini, L., Braghetta, P., Columbaro, M., Volpin, D., Bressan, G.M., et al. (2003). Mitochondrial dysfunction and apoptosis in myopathic mice with collagen VI deficiency. Nat. Genet. *35*, 367–371.

Landes, T., Emorine, L.J., Courilleau, D., Rojo, M., Belenguer, P., and Arnauné-Pelloquin, L. (2010). The BH3-only Bnip3 binds to the dynamin Opa1 to promote mitochondrial fragmentation and apoptosis by distinct mechanisms. EMBO Rep. *11*, 459–465.

Martinou, I., Desagher, S., Eskes, R., Antonsson, B., André, E., Fakan, S., and Martinou, J.C. (1999). The release of cytochrome c from mitochondria during apoptosis of NGF-deprived sympathetic neurons is a reversible event. J. Cell Biol. *144*, 883–889.

Murphy, E., and Steenbergen, C. (2008). Mechanisms underlying acute protection from cardiac ischemia-reperfusion injury. Physiol. Rev. *88*, 581–609.

Ogasawara, J., Watanabe-Fukunaga, R., Adachi, M., Matsuzawa, A., Kasugai, T., Kitamura, Y., Itoh, N., Suda, T., and Nagata, S. (1993). Lethal effect of the anti-Fas antibody in mice. Nature *364*, 806–809.

Orogo, A.M., and Gustafsson, A.B. (2013). Cell death in the myocardium: my heart won't go on. IUBMB Life *65*, 651–656.

Palmer, C.S., Osellame, L.D., Laine, D., Koutsopoulos, O.S., Frazier, A.E., and Ryan, M.T. (2011). MiD49 and MiD51, new components of the mitochondrial fission machinery. EMBO Rep. *12*, 565–573.

Pena, J.C., Rudin, C.M., and Thompson, C.B. (1998). A Bcl-xL transgene promotes malignant conversion of chemically initiated skin papillomas. Cancer Res. *58*, 2111–2116.

Powers, S.K., Kavazis, A.N., and McClung, J.M. (2007). Oxidative stress and disuse muscle atrophy. J. Appl. Physiol. *102*, 2389–2397.

Rizzuto, R., Bernardi, P., and Pozzan, T. (2000). Mitochondria as all-round players of the calcium game. J. Physiol. *529*, 37–47.

Rodriguez, I., Matsuura, K., Khatib, K., Reed, J.C., Nagata, S., and Vassalli, P. (1996). A bcl-2 transgene expressed in hepatocytes protects mice from fulminant liver destruction but not from rapid death induced by anti-Fas antibody injection. J. Exp. Med. *183*, 1031–1036.

Romanello, V., Guadagnin, E., Gomes, L., Roder, I., Sandri, C., Petersen, Y., Milan, G., Masiero, E., Del Piccolo, P., Foretz, M., et al. (2010). Mitochondrial fission and remodelling contributes to muscle atrophy. EMBO J. *29*, 1774–1785.

Sandri, M. (2008). Signaling in muscle atrophy and hypertrophy. Physiology (Bethesda) *23*, 160–170.

Sandri, M. (2013). Protein breakdown in muscle wasting: role of autophagy-lysosome and ubiquitin-proteasome. Int. J. Biochem. Cell Biol. *45*, 2121–2129.

Sandri, M., Lin, J., Handschin, C., Yang, W., Arany, Z.P., Lecker, S.H., Goldberg, A.L., and Spiegelman, B.M. (2006). PGC-1alpha protects skeletal muscle from atrophy by suppressing FoxO3 action and atrophy-specific gene transcription. Proc. Natl. Acad. Sci. USA *103*, 16260–16265.

Santel, A., Frank, S., Gaume, B., Herrler, M., Youle, R.J., and Fuller, M.T. (2003). Mitofusin-1 protein is a generally expressed mediator of mitochondrial fusion in mammalian cells. J. Cell Sci. *116*, 2763–2774.

Schägger, H. (1995). Native electrophoresis for isolation of mitochondrial oxidative phosphorylation protein complexes. Methods Enzymol. *260*, 190–202.

Sciacco, M., and Bonilla, E. (1996). Cytochemistry and immunocytochemistry of mitochondria in tissue sections. Methods Enzymol. *264*, 509–521.

Scorrano, L., Ashiya, M., Buttle, K., Weiler, S., Oakes, S.A., Mannella, C.A., and Korsmeyer, S.J. (2002). A distinct pathway remodels mitochondrial cristae and mobilizes cytochrome c during apoptosis. Dev. Cell *2*, 55–67.

Seo, A.Y., Joseph, A.M., Dutta, D., Hwang, J.C.Y., Aris, J.P., and Leeuwenburgh, C. (2010). New insights into the role of mitochondria in aging: mitochondrial dynamics and more. J. Cell Sci. *123*, 2533–2542.

Siu, P.M., and Alway, S.E. (2005). Mitochondria-associated apoptotic signalling in denervated rat skeletal muscle. J. Physiol. *565*, 309–323.

Wang, X. (2001). The expanding role of mitochondria in apoptosis. Genes Dev. *15*, 2922–2933.

Wang, Z., Jiang, H., Chen, S., Du, F., and Wang, X. (2012). The mitochondrial phosphatase PGAM5 functions at the convergence point of multiple necrotic death pathways. Cell *148*, 228–243.

Wasilewski, M., and Scorrano, L. (2009). The changing shape of mitochondrial apoptosis. Trends Endocrinol. Metab. *20*, 287–294.

Wei, M.C., Lindsten, T., Mootha, V.K., Weiler, S., Gross, A., Ashiya, M., Thompson, C.B., and Korsmeyer, S.J. (2000). tBID, a membrane-targeted death ligand, oligomerizes BAK to release cytochrome c. Genes Dev. *14*, 2060–2071.

Wei, M.C., Zong, W.X., Cheng, E.H., Lindsten, T., Panoutsakopoulou, V., Ross, A.J., Roth, K.A., MacGregor, G.R., Thompson, C.B., and Korsmeyer, S.J. (2001). Proapoptotic BAX and BAK: a requisite gateway to mitochondrial dysfunction and death. Science *292*, 727–730.

Yamaguchi, R., Lartigue, L., Perkins, G., Scott, R.T., Dixit, A., Kushnareva, Y., Kuwana, T., Ellisman, M.H., and Newmeyer, D.D. (2008). Opa1-mediated cristae opening is Bax/Bak and BH3 dependent, required for apoptosis, and independent of Bak oligomerization. Mol. Cell *31*, 557–569.

Yin, X.M., Wang, K., Gross, A., Zhao, Y., Zinkel, S., Klocke, B., Roth, K.A., and Korsmeyer, S.J. (1999). Bid-deficient mice are resistant to Fas-induced hepatocellular apoptosis. Nature *400*, 886–891.

Zhang, Z., Wakabayashi, N., Wakabayashi, J., Tamura, Y., Song, W.J., Sereda, S., Clerc, P., Polster, B.M., Aja, S.M., Pletnikov, M.V., et al. (2011). The dynamin-related GTPase Opa1 is required for glucose-stimulated ATP production in pancreatic beta cells. Mol. Biol. Cell *22*, 2235–2245.

Cell Metabolism
Supplemental Information

The Opa1-Dependent Mitochondrial Cristae Remodeling Pathway Controls Atrophic, Apoptotic, and Ischemic Tissue Damage

Tatiana Varanita, Maria Eugenia Soriano, Vanina Romanello, Tania Zaglia, Rubén Quintana-Cabrera, Martina Semenzato, Roberta Menabò, Veronica Costa, Gabriele Civiletto, Paola Pesce, Carlo Viscomi, Massimo Zeviani, Fabio Di Lisa, Marco Mongillo, Marco Sandri, and Luca Scorrano

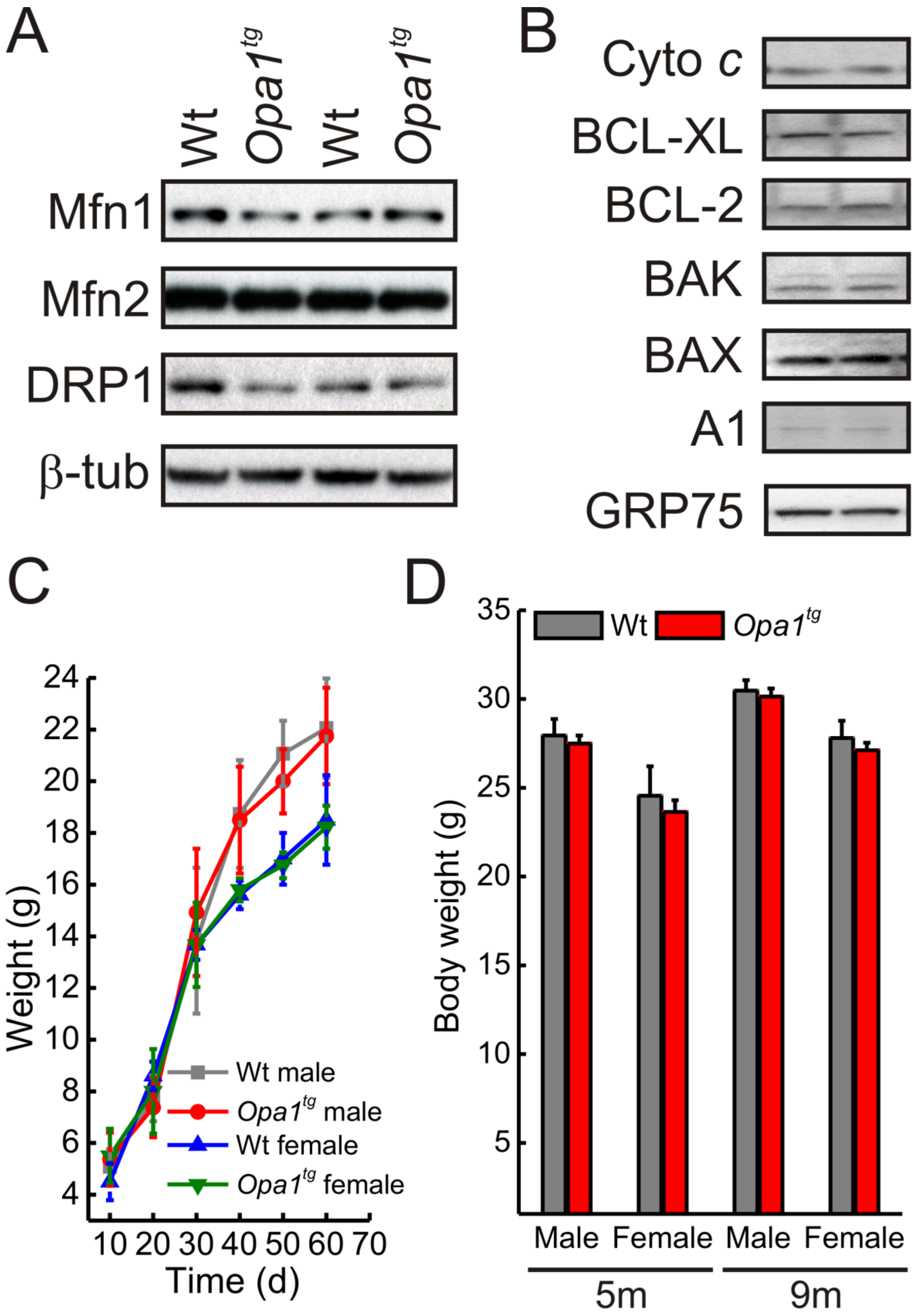

Supplemental Figure 1. Levels of mitochondria-shaping and apoptosis proteins are not changed in *Opa1^{tg}* Mice, Related to Fig. 1

(A) Equal amounts of protein from liver tissue of the indicated genotypes were separated by SDS-PAGE and immunoblotted with the indicated antibodies.

(B) Equal amounts of protein from liver tissue of the indicated genotypes were separated by SDS-PAGE and immunoblotted with the indicated antibodies.

(C) Body weight of C57/Bl6 individuals of the indicated genotype and sex for the indicated time. Data represent average + SEM (n=20 per each group).

(D) Body weight is represented as average + SEM of 5 month males (n=19 for each group) and females (n= 12 for each group) and 9 month old males (n= 32 for each group) and females (n=18 for each group) Sv129 littermates of the indicated genotype.

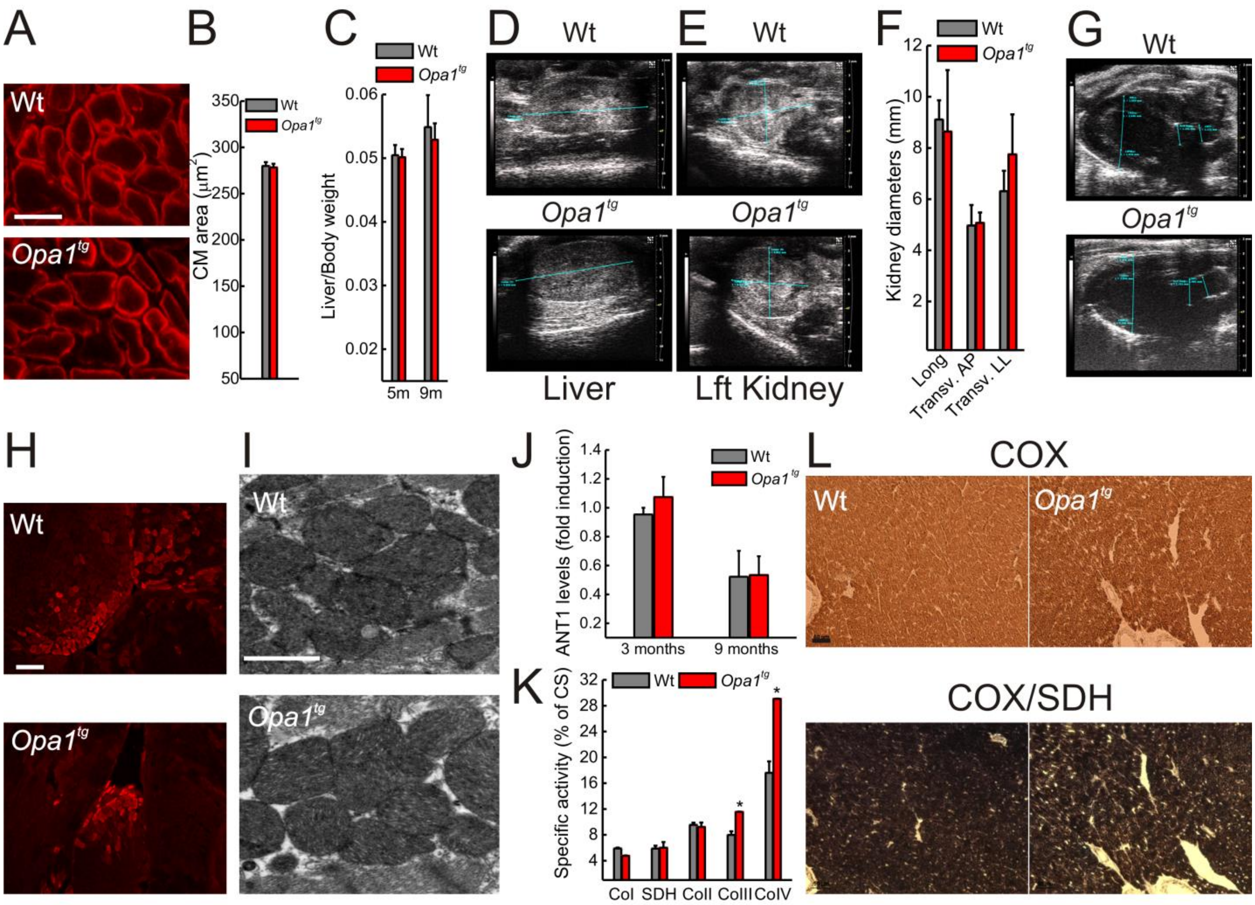

Supplemental Figure 2. Histological and Morphological Characterization of *Opa1*^{tg} Heart, Liver and Kidney, Related to Fig. 2

(A) Immunofluorescence analysis on ventricular cryosections from mice of the indicated genotype stained with an antibody to dystrophin. Images are details from the left ventricle. Scale bar, 25 µm.

(B) Quantification of cardiomyocyte (CM) area in cryosections from 5 month old hearts of the indicated genotype in experiments as in (A). Data are mean±SD of 5 individuals for each group).

(C) Quantification of liver weight/body weight ratio in 5 and 9 month old male mice of the indicated genotypes. Data represent average + SEM (n=10 for each group).

(D) Echography images of livers from 9 month old mice of the indicated genotype.

(E) Echography short axis view of the left kidney from 9 month old mice of the indicated genotype.

(F) Morphometric analysis of kidney from 9 month old mice of the indicated genotype in experiments as in (E). Data are mean±SD of 6 individuals in each group)

(G) Echocardiographic long axis view of hearts from 5 month old littermates of the indicated genotype. LV: left ventricle; A: aorta.

(H) Representative confocal images of ventricular cryosections from mice stained for beta-myosin heavy chain (β-MHC). Scale bar, 100 µm.

(I) Electron micrographs of hearts from 9 month old littermates of the indicated genotyopes. Scale bar, 1µm.

(J) Data represent average±SEM of the ANT1 mRNA levels determined by RT-PCR in hearts from littermates of the indicated genotype of the indicated age (n=3 for each group).

(K) Mitochondrial respiratory chain specific activities measured in hearts of the indicated genotypes normalized to that of citrate synthase. Data are average±SEM of 3 individuals for each group. *, p<0.05 in an unpaired two-sample Student's t-tests.

(L). Representative images of COX and double COX/SDH histological staining in heart sections from 9 month old littermates of the indicated genotypes. Scale bar, 50 µm.

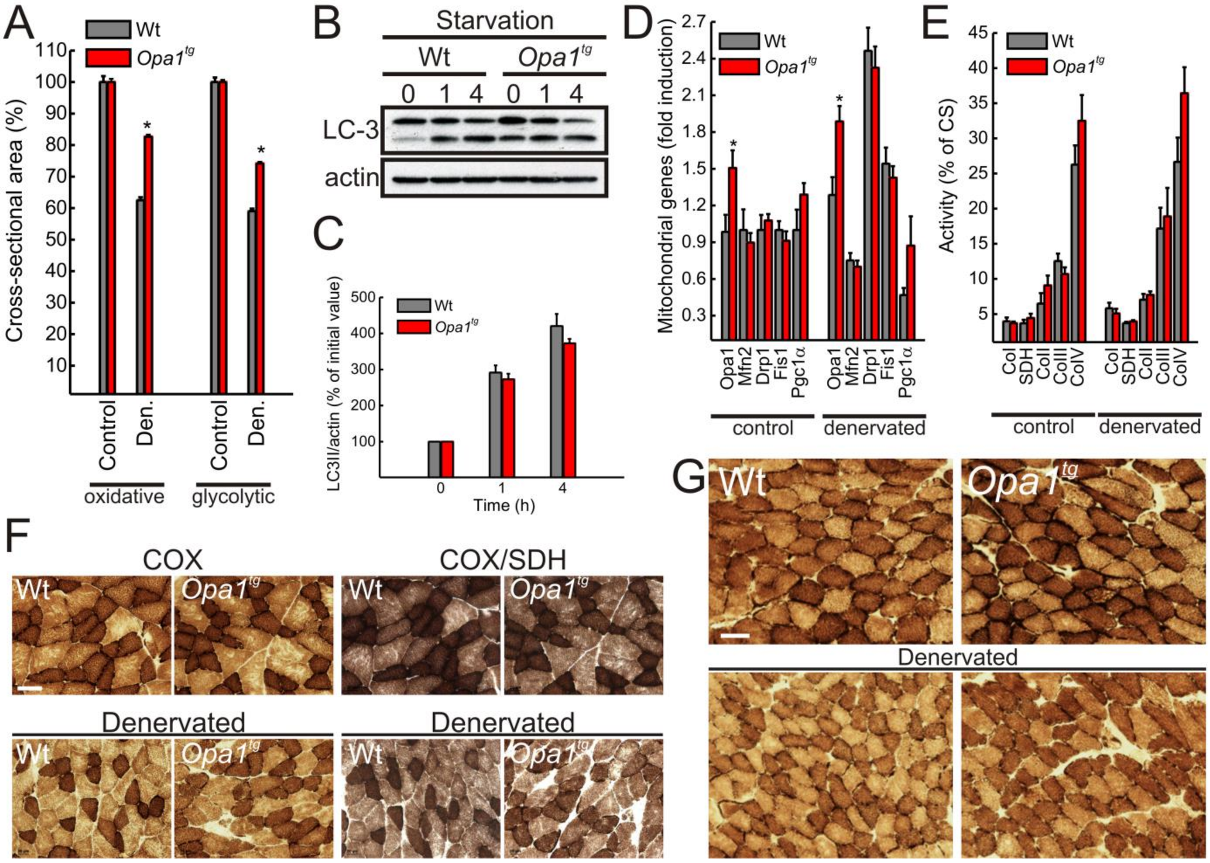

Supplemental Figure 3. Opa1^{tg} mice are protected from denervation induced muscular atrophy, related to Figure 3

(A) Cross-sectional area (CSA) measurements of innervated and denervated fibers of the indicated type 10 days after denervation. Data are average±SEM of 5 independent experiments. *, p<0.05 in an unpaired two-sample Student's t-tests.

(B) Autophagy was induced in mouse adult fibroblasts (MAFs) of the indicated genotypes by starvation in the presence of 100µM chloroquine, cells were lysed at the indicated time points and equal amounts of proteins were separated by SDS-PAGE and immunoblotted using the indicated antibodies.

(C) Densitometric analysis of experiments as in (B). Data represent average±SEM of 5 independent experiments and are normalized to the initial value.

(D) Data represent average±SEM of the indicated mRNA levels determined by RT-PCR in littermates of the indicated genotype treated as indicated (n=5 for each group). *, p<0.05 in an unpaired two-sample Student's t-tests.

(E) Mitochondrial respiratory chain specific activities measured in muscles of the indicated genotypes treated as indicated normalized to that of citrate synthase. Data are average±SEM of 5 individuals for each group. *, p<0.05 in an unpaired two-sample Student's t-tests.

(F, G) Representative images of COX and COX/SDH histological staining in control and denervated gastrocnemius (F) and soleus (G) muscles from littermates of the indicated genotypes treated as indicated. Scale bars, 50µm.

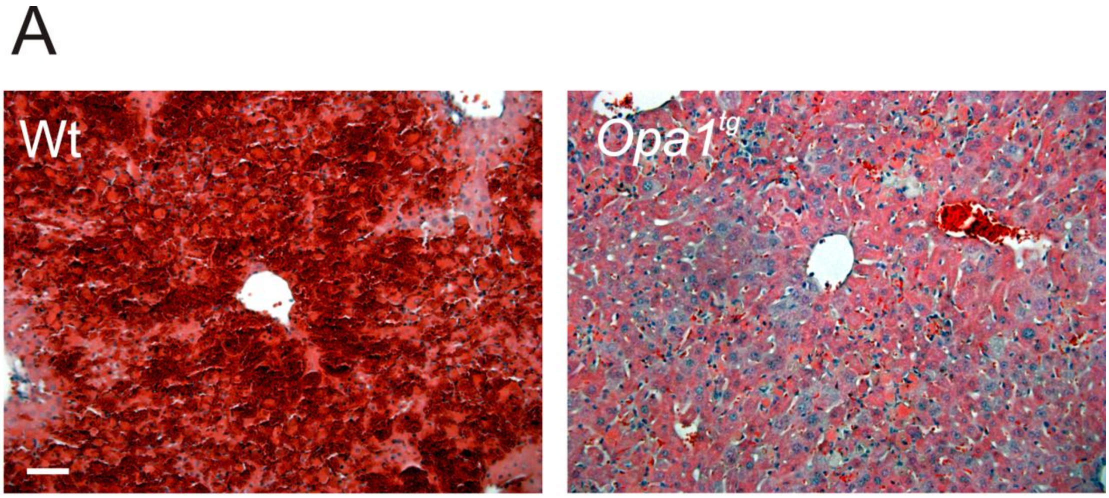

B

Genotype	Dead/injected
Wt	3/4
Opa1^{tg}	2/4

Supplemental Figure 4. Sv129 *Opa1^{tg}* Livers are ProtectedF rom Fas-Induced Damage, Related to Fig. 5

(A) Representative images of Hematoxylin-Eosin stained paraffin embedded liver sections from littermates of the indicated genotype tail vein injected with 0.25µg/g anti-Fas antibody (Fas) and sacrificed after 24h. Scale bar, 50 µm.

(B) Number of dead Sv129 mice of the indicated genotype 24 h after tail vein injection of 0.25µg/g anti-Fas antibody

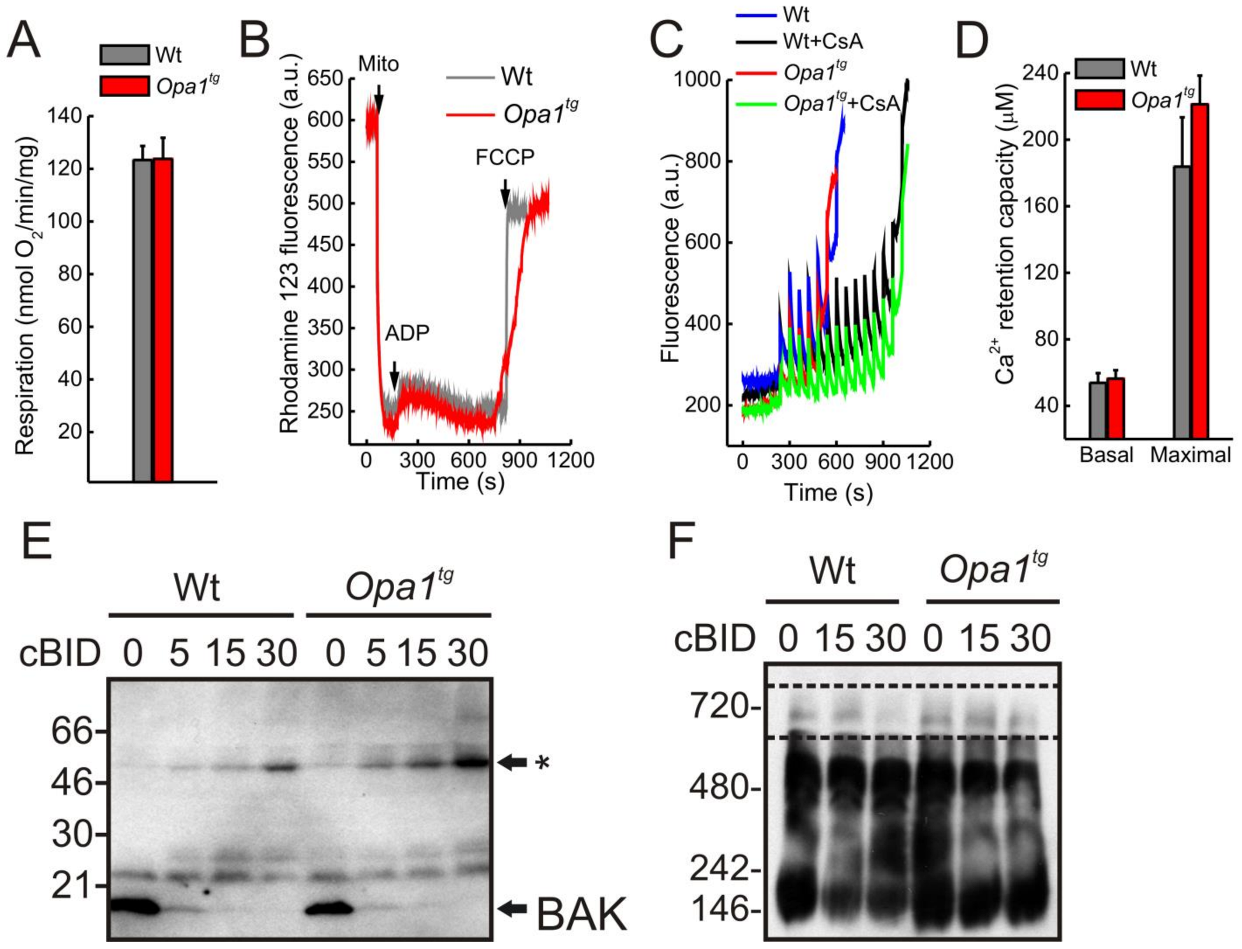

Supplemental Figure 5. *Opa1^{tg}* Mitochondrial Function Is Not Altered, Related to Figure 6

(A) Complex IV dependent respiration of mitochondria isolated from livers of the indicated genotypes. Data represent average $\pm$ SEM (n=4 for each group).

(B) Representative traces of Rhodamine 123 fluorescence. Mitochondria isolated from livers of the indicated genotype (1 mg/mL, Mito) were treated where indicated (arrows) with 300 μM ADP and 200 nM FCCP.

(C) Representative traces of Calcium Retention Capacity (CRC) of liver mitochondria isolated from 5 month old mice of the indicated genotype. For the assessment of the maximal CRC, 2 μM Cyclosporine A (CsA) was present in the medium.

(D) Quantification of CRC in experiments as in (C).

(E) Mitochondria of the indicated genotype were treated with cBID for the indicated times, crosslinked with. 10 mM BMH and after 30 min the crosslinking reaction was quenched. Equal amounts (40μg) of proteins were analyzed by SDS-PAGE/immunoblotting using anti-BAK antibody. Asterisks:BAK multimers.

(F) Representative BNGE analysis of OPA1 oligomers in muscle mitochondria of the indicated genotypes treated for the indicated times with cBID. The boxed area indicates the OPA1 high molecular weight complexes.

Supplemental Experimental Procedures

Ecocardiography

Two-dimensional cine loops with frame rates of 200 frames/s of a long-axis view and a short-axis view at proximal-, mid- and apical level of the left ventricle (LV) were recorded. Inter ventricular septum (IVS) and LV posterior wall (LVPW) thicknesses, LV internal diameter (LVID) and maximal LV length were measured in systole (s) and in diastole (d) from the long axis B-mode image, according to standard procedures. Ejection fraction (EF) was determined by the following formula: Simp EF (%) = 100 x Simp Systolic Volume (SV)/ Simp LV volume; d. To assess changes in LV shape, the sphericity index (SI) was calculated at end-diastole (d) and end-systole (s) as the volume of the LV divided by the volume of a sphere with a diameter equal to the LV longest axis (measured in the apical view) using the following formulas: SI: d=LV volume; d/(3/4*Π*(Simp Length; d)^3); SI: s = LV volume; s/(3/4*Π*(Simp Length; s)^3).

As this ratio increases, the ventricle becomes more spherical. Echocardiographic image acquisition and analysis were performed by a single operator, blinded to the mouse genotype.

Gene expression analyses

The following oligonucleotides primers were employed:

Opa1 forward 5'-ATACTGGGATCTGCTGTTGG-3' reverse 5'-AAGTCAGGCACAATCCACTT; *Drp1* forward 5'-TCAGATCGTCGTAGTGGGAA reverse 5'-TCTTCTGGTGAAACGTGGAC-3'; *Fis1* forward 5'-AAGTATGTGCGAGGGCTGT-3' reverse 5'-TGCCTACCAGTCCATCTTTC-3'; *Mfn2* forward 5'-ATGTTACCACGGAGCTGGAC-3' reverse 5'-AACTGCTTCTCCGTCTGCAT-3'; *Pgc1a1* forward 5'-GGAATGCACCGTAAATCTGC-3' reverse 5'-TTCTCAAGAGCAGCGAAAGC-3'; *Atrogin-1* forward 5'-GCAAACACTGCCACATTCTCTC-3' reverse 5'-CTTGAGGGGAAAGTGAGACG-3'; *MuRF-1* forward 5'-

ACCTGCTGGTGGAAAACATC-3’ reverse 5’-ACCTGCTGGTGGAAAACATC-3’; *Musa1* forward 5’-TCGTGGAATGGTAATCTTGC-3’ reverse 5’-CCTCCCGTTTCTCTATCACG-3’; *Bnip3* forward 5’-TTCCACTAGCACCTTCTGATGA-3’ reverse 5’-GAACACCGCATTTACAGAACAA-3’; *CathepsinL* forward 5’-GTGGACTGTTCTCACGCTCAAG-3’ reverse 5’-TCCGTCCTTCGCTTCATAGG-3’; *FoxO1* forward 5’-GCTGGGTGTCAGGCTAAGAG-3’ reverse 5’-GAGGGGTGAAGGGCATCT-3’; *FoxO3* forward 5’-CGCTGTGTGCCCTACTTCA-3’ reverse 5’-CCCGTGCCTTCATTCTGA-3’; *FoxO4* forward 5’-CGGAGTGAAAGGGACAGTTTAG-3’ reverse 5’-CCCTGTGGCTGACTTCTTATTC-3’; *LC3b* forward 5’-CACTGCTCTGTCTTGTGTAGGTTG-3’ reverse 5’-TCGTTGTGCCTTTATTAGTGCATC-3’; *p62* forward 5’-CCCAGTGTCTTGGCATTCTT-3’ reverse 5’-AGGGAAAGCAGAGGAAGCTC-3’; *ActR2B* forward 5’-TTAAGGATCACTGGCTGAAACA-3’ reverse 5’-GGATACCCGCTCTTCTACACAG-3’; *Myostatin* forward 5’-AGTAAAAGCCCAACTGTGGATA-3’ reverse 5’-TCATGTCAAGTTTCAGAGATCG-3’; *Alk3* forward 5’-CTGGGAGTGGATCTGGATTG-3’ reverse 5’-CTGTTTGGCAATAGTTCGCTG-3’; *Gapdh* forward 5’-CACCATCTTCCAGGAGCGAG-3’ reverse 5’-CCTTCTCCATGGTGGTGAAGAC-3’; *Mul1* forward 5’- AGGGCATTCTTTCAGAAGCA-3’ reverse 5’-GGGGTGGAACTTCTCGTACA-3’; *Actin* 5’-forward 5’-CTGGCTCCTAGCACCATGAAGAT-3’ reverse 5’-GGTGGACAGTGAGGCCAGGAT-3’; *Anp* forward 5’-GCAGAGACAGCAAACATCAGA-3’ reverse 5’-GCATCTTCTCCTCCAGGTG-3’.

Biochemistry

The following primary antibodies were used: Monoclonal anti-OPA1 (1:1000 BD), rabbit polyclonal anti-MFN1 (1:1000 Millipore), monoclonal anti-MFN2 (1:1000 Abnova), monoclonal anti-DLP1 (1:1000 BD), monoclonal anti-Cytochrome c (1:1000 BD Pharmingen), rabbit polyclonal anti-BCL-XL (1:1000 Santa Cruz Biotechnology) , rabbit polyclonal anti-BCL-2 (1:1000 Santa Cruz Biotechnology), rabbit polyclonal anti-BAK-NT (1:1000 Upstate), rabbit polyclonal

anti-BAX (1:1000 Millipore), rabbit polyclonal anti-GRP75 (1:1000 Santa Cruz Biotechnology), rabbit polyclonal anti-A1 (1:1000 Santa Cruz Biotechnology), monoclonal anti-ACTIN (1:1000 Chemicon), monoclonal anti-CII (1:2000 Abcam).

Autophagy analysis

Autophagy was induced by serum starvation. Cells of the indicated genotypes were washed 4 times and then incubated in Earle's Balanced Salt Solution (EBSS) at 37°C for the indicated time. To block autophagic flux, 100 μM chloroquine (CQ) was added.

Immunohistochemistry

We used the following antibodies: rabbit anti-dystrophin (1:600 Abcam), rabbit anti-collagen I (1:800 Acris), rabbit anti-Atrial Natriuretic Peptide (ANP, 1:500 Peninsula Lab), monoclonal anti-cytochrome c (1:200 BD Pharmingen), rabbit polyclonal anti-TOM20 (1:200 Santa Cruz Biotechnology). To detect the primary antibodies the following secondary antibodies were used: Alexa Fluor 488, or 568 (1:200 Invitrogen), FITC and TRITC-conjugated secondary antibodies (Jackson Labs).

Assays of mitochondrial membrane potential

FDB myofibres were placed in 1ml Tyrode's buffer and loaded with 5 nM TMRM (Molecular Probes) supplemented with 1 μM cyclosporine H (a P-glycoprotein inhibitor) for 30 min at 37°C. Myofibres were then observed using an Olympus IX81 inverted microscope (Melville, NY) equipped with a CellR imaging system. Sequential images of TMRM fluorescence were acquired

every 60s with a 4X objective (Olympus). Where indicated, oligomycin (5nM, Sigma) or the uncoupler carbonyl cyanide p-trifluoromethoxyphenylhydrazone (FCCP, 400nM, Sigma) was added. TMRM fluorescence analysis over the mitochondrial regions of interest was performed using ImageJ.

In vitro mitochondrial assays

Mitochondria Ca^{2+} retention capacity was measured fluorimetrically using a Perkin Elmer LS50B fluorimeter (λ_{ex} 505 nm; λ_{em}: 535 nm). Freshly isolated mitochondria were resuspended in experimental buffer (EB) (Frezza et al., 2007) supplemented with 5mM glutamate/2.5mM malate and 1μM Calcium Green-5N (Invitrogen). Pulses of 10μM Ca^{2+} were added every 30 seconds until PTP induction. For the maximal CRC, EB was supplemented with 2μM Cyclosporine A.

To measure mitochondrial membrane potential EB was supplemented with 0.2μM Rhodamine 123 and fluorescence was measured using a Perkin Elmer LS50B fluorimeter (λ_{ex} 480 nm; λ_{em}: 540 nm)

Morphometric analysis

Cardiomyocyte cross-sectional area was quantified in five non-consecutive cryosections from the mid-portion of the ventricles per heart were stained with an antibody specific for dystrophin and analyzed by fluorescence microscopy. The images of twelve randomly chosen fields of 69000 μm^2 of area from the ventricles were acquired with a fluorescence microscope (Leica DC130, Leica Microscopes, Germany) and cardiomyocyte cross-sectional area was evaluated using the software ImageJ.

Fiber Cross-Sectional Area (CSA) was measured using ImageJ. More than 1000 fibers per each muscle were counted. The fields were randomly selected to measure the fiber area and all the fibers encompassed in those fields were evaluated. This analysis was carried out in a blind fashion, with the operator unaware of the mice genotype.

Mitochondrial length was measured using the ImageJ Plugin *Mosaic Squassh* (Rizk et al., 2014)

Imaging

Hematoxylin and eosin (H&E), COX/SDH and TUNEL were performed on paraffin embedded sections or cryosections, immunofluorescence was performed on cryosections as previously described. H&E, COX/SDH and TUNEL were imaged with a bright field on fluorescence microscope (Leica DM 5000B) using 20x or 40x PlanApo objectives (Leica). Confocal images were acquired using a confocal microscope (TCS SP5; Leica) using the LAS AF software (Leica), a 63×, 1.4 NA objective, and the 488-nm and 561-nm laser lines.

Supplemental References

Frezza,C., Cipolat,S., and Scorrano,L. (2007). Organelle isolation: functional mitochondria from mouse liver, muscle and cultured fibroblasts. Nat. Protoc. 2, 287-295.

Rizk,A., Paul,G., Incardona,P., Bugarski,M., Mansouri,M., Niemann,A., Ziegler,U., Berger,P., and Sbalzarini,I.F. (2014). Segmentation and quantification of subcellular structures in fluorescence microscopy images using Squassh. Nature Protocols 9, 586-596.
